# Antioxidant and oxidative enzymes, genetic variants, and cofactors as prognostic biomarkers of COVID-19 severity and mortality: a systematic review

**DOI:** 10.3389/fmolb.2025.1700263

**Published:** 2025-10-23

**Authors:** Shukur Wasman Smail, Blnd Azad Ismail, Ivan Sdiq Maghdid, Abdullah Hayder Flaih, Christer Janson

**Affiliations:** ^1^ College of Pharmacy, Cihan University-Erbil, Erbil, Iraq; ^2^ Department of Biology, College of Science, Salahaddin University-Erbil, Erbil, Iraq; ^3^ Department of Medical Science, Respiratory Medicine, and Allergology, Uppsala University and University Hospital, Uppsala, Sweden

**Keywords:** COVID-19, oxidative stress, antioxidant enzymes, genetic polymorphisms, prognostic biomarkers

## Abstract

Oxidative stress plays a pivotal role in the pathogenesis and progression of coronavirus disease 2019 (COVID-19). Severe acute respiratory syndrome coronavirus 2 (SARS-CoV-2) infection disrupts redox homeostasis through excessive generation of reactive oxygen and nitrogen species, driving inflammation, endothelial dysfunction, and multi-organ injury. Serum oxidative and antioxidative enzymes, their genetic polymorphisms, and essential micronutrient cofactors have emerged as potential prognostic biomarkers for COVID-19 severity and mortality. Evidence indicates that imbalances in antioxidant enzymes such as superoxide dismutase, catalase, glutathione peroxidase, and glutathione reductase correlate with disease progression, while polymorphisms in GST, superoxide dismutase, CAT, and HO-1 genes may modify susceptibility and outcomes. Biomarkers of oxidative damage, including malondialdehyde, 8-isoprostanes, nitrotyrosine, and protein carbonyls, consistently associate with respiratory failure, intensive care admission, and mortality. Furthermore, micronutrients such as selenium, zinc, copper, manganese, and iron, which act as enzymatic cofactors, influence antioxidant defense capacity and clinical prognosis. Despite promising data, limitations in biomarker standardization and assay specificity remain key challenges for clinical translation. The aim of this systematic review is to integrate enzymatic, genetic, and cofactor-based biomarkers to enhance risk stratification, challenging and to improve prognostic modelling in COVID-19. A better understanding of these biomarkers may facilitate early identification of high-risk patients, guide therapeutic interventions, and ultimately improve clinical outcomes in COVID-19.

## 1 Introduction

Since its emergence in late 2019, coronavirus disease 2019 (COVID-19) has remained a global health crisis, with severity ranging from mild respiratory illness to acute respiratory distress syndrome (ARDS), multi-organ failure, and death. Mounting evidence highlights the role of oxidative stress (OS) in this spectrum, with viral replication, mitochondrial dysfunction, and inflammatory cascades collectively driving excessive production of reactive oxygen species (ROS) (Gain et al., 2022). This redox imbalance contributes to endothelial injury, cytokine storm, thrombosis, and long-term complications in survivors (Cusato et al., 2023).

Under physiological conditions, antioxidant enzymes such as superoxide dismutase (SOD), catalase (CAT), glutathione peroxidase (GPx), and glutathione reductase (GR) form the first line of defence against ROS, with essential micronutrients—including selenium (Se), zinc (Zn), copper (Cu), and manganese (Mn)—acting as indispensable cofactors (Birk, 2021). However, in the context of severe acute respiratory syndrome coronavirus 2 (SARS-CoV-2) infection, nutrient deficiencies and increased oxidative load compromise enzymatic defences, amplifying tissue damage. Adding to this complexity, genetic polymorphisms in antioxidant enzyme genes modulate susceptibility and outcomes. Variants such as SOD2 rs4880, GSTP1, GSTO1, PON1, and NOS3 have been associated with reduced enzymatic activity, impaired detoxification of ROS, and worsened COVID-19 severity or post-acute sequelae (Eid et al., 2024).

Micronutrients further influence host response beyond their role as cofactors. For example, selenium deficiency has been linked to higher mortality in COVID-19, owing to impaired immune and antioxidant functions (Mohammadi et al., 2023; Renata et al., 2023). Such findings highlight the need for an integrative approach that considers enzymatic, genetic, and nutritional determinants in understanding COVID-19 outcomes.

Despite rapid advances in understanding COVID-19, there remains a pressing need for reliable prognostic biomarkers that can stratify patients by risk and guide targeted interventions. Current clinical biomarkers, such as inflammatory cytokines and D-dimer, provide only partial insight into disease dynamics. By contrast, OS–related enzymes, their genetic polymorphisms, and micronutrient cofactors offer a multidimensional perspective that links viral pathophysiology with host susceptibility. Importantly, these biomarkers are measurable, mechanistically relevant, and potentially modifiable through nutritional, pharmacological, or genetic interventions. To date, no comprehensive synthesis has integrated these three layers—biochemical, genetic, and nutritional—within the context of COVID-19. This systematic review synthesizes current evidence on antioxidant and oxidative enzymes, genetic variants, and metabolic cofactors as prognostic biomarkers of COVID-19 severity and mortality, aiming to establish an integrated framework to support precision prognostication and improve infectious disease management.

## 2 Methodology

### 2.1 Databases and time frame

We systematically searched PubMed/MEDLINE, Scopus, Web of Science Core Collection, and Embase for studies published 1 December 2019 to 20 September 2025. To ensure completeness, we screened reference lists of included articles and relevant reviews. Preprints in medRxiv/bioRxiv were checked for peer-reviewed versions; only peer-reviewed articles were included in the main synthesis.

#### 2.1.1 Scope of pre-2019 literature

Our primary evidence base for clinical COVID-19 associations was restricted to studies published 1 December 2019–20 September 2025. We also included pre-2019 publications when they provided foundational context essential to interpret COVID-19 biomarkers, specifically: (i) mechanistic or reference texts on oxidative and nitrosative stress biology and redox signalling; (ii) assay standardization/analytical methods (e.g., Griess reaction, lipid peroxidation biomarkers); and (iii) enzyme and genetic background (e.g., GST/SOD/HO-1 biology, population genetics, nomenclature). These pre-2019 sources were not used as primary evidence for COVID-19 prognosis/diagnosis but to contextualize COVID-19 findings and justify biomarker selection. Conclusions about severity, ICU admission, and mortality rely on the 2019–2025 COVID-19 clinical literature.

### 2.2 Search strategy

Database-specific Boolean strings combined controlled vocabulary (e.g., MeSH, Emtree) and free-text terms. Core keywords included: SNP, prognosis, diagnostic biomarker, severity, mortality, COVID-19, SARS-CoV-2.

### 2.3 Eligibility criteria

#### 2.3.1 Inclusion

Human studies with confirmed COVID-19; 2) report at least one oxidative-stress–related biomarker (enzymatic activity, e.g., SOD, catalase, GPX, HO-1; genetic variants/SNPs, e.g., GSTM1/GSTT1, GSTP1, SOD2 rs4880, HMOX1, NOS3; micronutrient cofactors: selenium, zinc, copper, manganese, iron; oxidative damage biomarkers—MDA, 8-isoprostanes, nitrotyrosine, protein carbonyls, 4-HNE, 8-OHdG; and oxidative-stress–responsive miRNAs, e.g., miR-21, miR-146a, miR-155); 3) examine clinical relevance (diagnosis, severity, ICU admission, ventilation, mortality, or prognostic modelling).

#### 2.3.2 Exclusion

Case reports/series n < 5, editorials, narrative opinions, non-COVID-19 cohorts, *in vitro* studies without translational linkage, unclear biomarker methods, or absence of clinically relevant outcomes. Where duplicate cohorts were suspected, the most comprehensive/updated report was retained.

### 2.4 Study selection and flow

**FIGURE 1:**
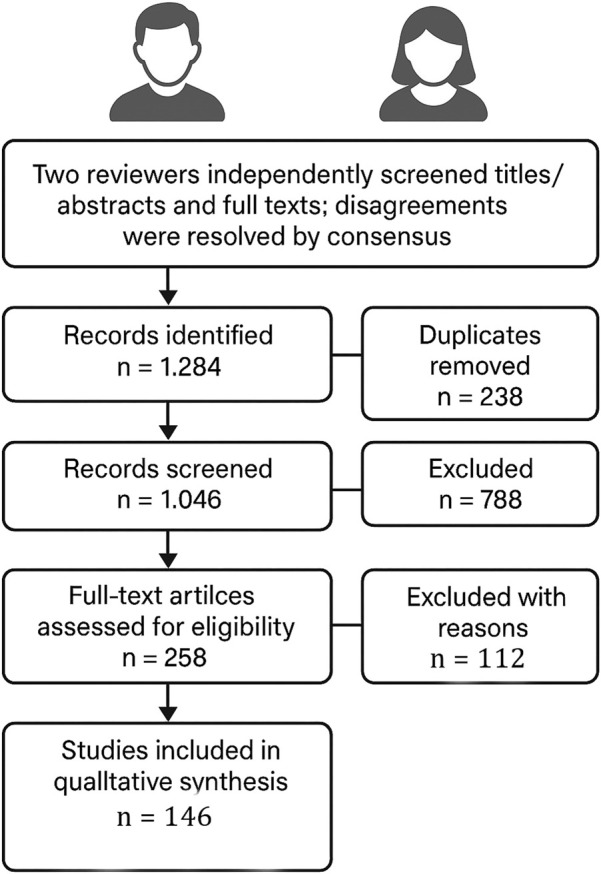
PRISMA flow diagram illustrating the study selection process.

Two reviewers independently screened titles/abstracts and full texts; disagreements were resolved by consensus. The search yielded 1,284 records; after deduplication (n = 238), 1,046 records were screened, 788 excluded by title/abstract, and 258 full texts assessed. In this systematic review, 146 studies met the inclusion criteria and were synthesized qualitatively; the remaining 112 full-text articles were excluded with reasons (Figure 1).

### 2.5 Data extraction

We extracted: study design, country/setting, sample size, patient characteristics, viral variant era (when reported), biomarker(s) measured (analyte, matrix, assay platform, timing), comparator groups, clinical endpoints (diagnosis, severity scales, ICU, ventilation, mortality), and effect estimates (e.g., OR/HR, AUC), plus adjustment covariates.

### 2.6 Quality and risk-of-bias assessment

Observational prognostic/association studies were appraised using QUIPS (risk of bias in prognostic factor studies) or the Newcastle–Ottawa Scale; genetic association studies were additionally considered against HuGENet/Q-Genie domains (selection, comparability, genotyping quality, HWE, multiple testing). We noted assay standardization issues (e.g., variability in MDA, 8-isoprostane, nitrotyrosine, and nitrate/nitrite methods) and timing of sampling as potential bias sources.

### 2.7 Synthesis approach

Given heterogeneity in populations, sampling time points, and assays, we conducted a narrative synthesis. To enhance interpretability (per reviewer request), biomarkers were stratified by strength of evidence:

• Strong: ≥3 independent cohorts with consistent directionality and adjusted associations with severity/ICU/mortality, or replicated diagnostic performance (e.g., HO-1, nitrate/nitrite (NOS activity), selenium deficiency, SOD2 rs4880, MDA/8-isoprostanes/nitrotyrosine, and miR-21/miR-146a).
• Inconsistent: mixed findings across cohorts or assay platforms (e.g., catalase, SOD activity, GSTM1/GSTT1, GSTP1, miR-155).
• Weak/Preliminary: limited studies or small samples without replication (e.g., MPO, XO, PRDX/TXN/TXNR/NQO1 variants, other miRNAs).

## 3 SARS-CoV-2 and COVID-19 pathogenesis

SARS-CoV-2 is an enveloped, positive-sense RNA virus of the Coronaviridae family. It enters host cells via binding of its spike (S) protein to angiotensin-converting enzyme 2 (ACE2), expressed in the respiratory tract, vascular endothelium, and gastrointestinal tissues. Viral entry downregulates ACE2 and disrupts the renin–angiotensin system (RAS), driving endothelial dysfunction, OS, and inflammation. The resulting clinical syndrome, COVID-19, ranges from asymptomatic infection to severe respiratory failure and multi-organ dysfunction (Cascella et al., 2020). COVID-19 presents in four clinical stages: Mild disease involves non-specific symptoms such as fever, cough, sore throat, headache, anosmia, and fatigue without hypoxemia or lung involvement. Moderate disease occurs when infection extends to the lower respiratory tract, causing pneumonia with pulmonary infiltrates and oxygen saturation (SpO_2_ ≥94%) but generally stable vital signs. Severe disease is defined by hypoxemia (SpO_2_ <94%), respiratory distress, and >50% lung involvement, often accompanied by systemic inflammation, OS, and complications such as ARDS, thromboembolism, and myocardial injury. The most advanced stage, Critical COVID-19, is marked by respiratory failure requiring mechanical ventilation, shock, and/or multi-organ dysfunction, with cytokine storm, profound OS, and hypercoagulability driving high mortality risk (Domínguez et al., 2020; Forrest et al., 2022).

### 3.1 Oxidative stress aspects of SARS-CoV-2

#### 3.1.1 Free radical and oxidative stress

**FIGURE 2:**
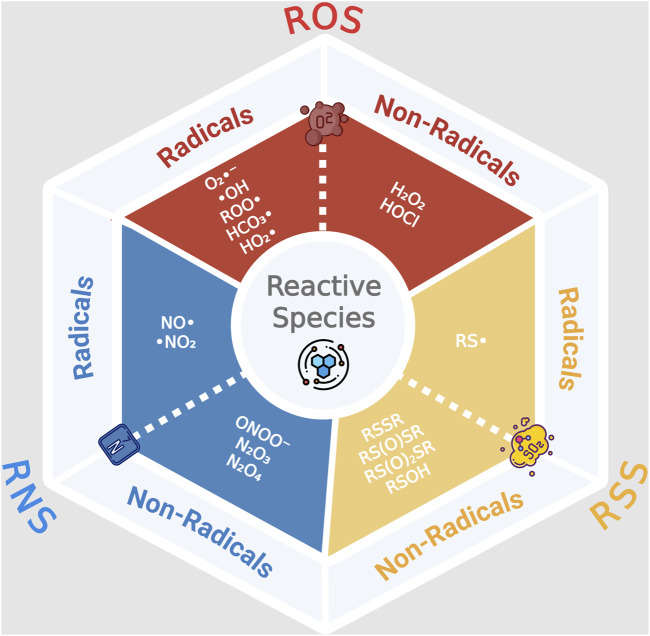
Classification of Reactive Species into Free Radicals and Non-Radicals across ROS, RNS, and RSS. The diagram illustrates the three major classes of reactive species: Reactive Oxygen Species (ROS, red), Reactive Nitrogen Species (RNS, blue), and Reactive Sulfur Species (RSS, yellow). Each class is further subdivided into radicals (species with unpaired electrons) and non-radicals (highly reactive but without unpaired electrons). Abbreviations: ROS, Reactive Oxygen Species; RNS, Reactive Nitrogen Species; RSS, Reactive Sulfur Species; O_2_•^-^, superoxide anion radical; •OH, hydroxyl radical; ROO•, peroxyl radical; HO_2_•, hydroperoxyl radical; NO•, nitric oxide radical; •NO_2_, nitrogen dioxide radical; RS•, thiyl radical; H_2_O_2_, hydrogen peroxide; HOCl, hypochlorous acid; ONOO^−^, peroxynitrite; N_2_O_3_, dinitrogen trioxide; N_2_O_4_, dinitrogen tetroxide; RSSR, disulfide; RSO_2_SR, sulfonyl-containing disulfides; RSOSR, sulfinyl derivative of thiols; RSOH, sulfenic acid. The image was created using the BioRender program, available at https://www.biorender.com.

OS is classically defined as an imbalance between oxidant production and antioxidant defenses, leading to oxidative molecular damage (Jones, 2006). A free radical is any chemical species, neutral or charged, with one or more unpaired electrons, making it highly unstable (Halliwell and Gutteridge, 2015). In aerobic organisms, the main reactive species are ROS (Halliwell and Gutteridge, 2015), but additional families include reactive nitrogen species (RNS) and reactive sulphur species (RSS), which interact with or enhance ROS generation (Figure 2).

### 3.2 The mechanism of SARS-CoV-2 induced oxidative stress

**FIGURE 3:**
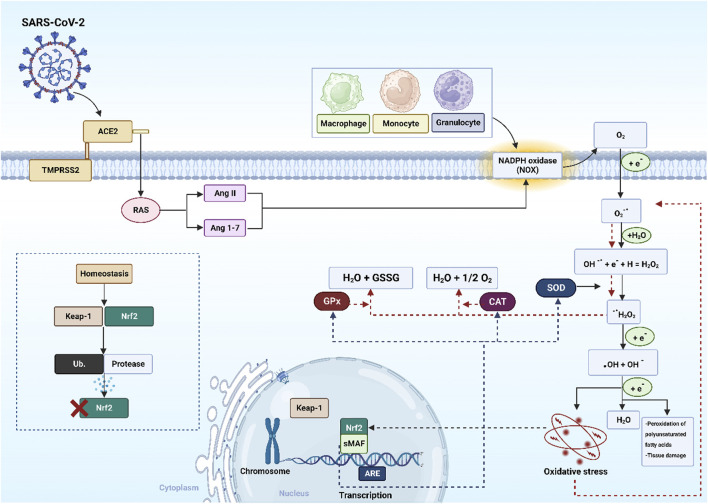
The mechanism of SARS-CoV-2 induced oxidative stress. Abbreviations: nrf2: nuclear factor erythroid 2-related factor 2, sMAF: small musculoaponeurotic fibrosarcoma, KEAP1: kelch-like ECH- associated protein 1, AREs: antioxidant response elements, RAS: renin-angiotensin system, SOD: superoxide dismutase, CAT: catalase, GPx glutathione peroxidase, SARS-CoV-2: severe acute respiratory syndrome-coronavirus-2, ACE2: angiotensin-converting enzyme 2, TMPRSS2: transmembrane serine protease 2, Ang: angiotensin, GSSG: oxidized glutathione, Ub: ubiquitination, H_2_O_2:_ hydrogen peroxide, H_2_O: water, •OH: hydroxyl radical, O_2_^−•^: superoxide anion, OH^−^: hydroxyl ion, e^−^: electron, O_2_: oxygen. The image was created using the BioRender program, available at https://www.biorender.com.

SARS-CoV-2 enters cells via ACE2 and transmembrane protease serine 2 (TMPRSS2), where it is detected by pattern recognition receptors (PRRs). This triggers immune cell recruitment, pro-inflammatory cytokine release, and elevated OS. The virus also disrupts RAS by activating NADPH oxidase (NOX) and increasing angiotensin (Ang) II, which drives superoxide anion (O_2_^−•^) generation. Mitochondrial electron transfer dysfunction further amplifies ROS production, including hydrogen peroxide (H_2_O_2)_ and hydroxyl radical (•OH) (Sies and Jones, 2020). During inflammation and OS, lipid peroxidation activates nuclear factor erythroid 2-related factor 2 (nrf2) and small musculoaponeurotic fibrosarcoma (sMAF), which translocates to the nucleus, binds antioxidant response elements (AREs), and upregulates antioxidant enzymes (SOD, CAT, GPx). However, excessive ROS/RNS in COVID-19 can suppress Nrf2, promoting apoptosis and lung cell death (Robledinos-Antón et al., 2019). Meanwhile, free radicals activate nuclear factor-κB (NF-кB), driving pro-inflammatory cytokine expression. Under homeostasis, Nrf2 is tightly regulated by kelch-like ECH- associated protein 1 (KEAP1), which promotes its ubiquitination (Ub) to prevent overactivation (Liu et al., 2017) as shown in (Figure 3). In COVID-19, SARS-CoV-2 binding downregulates ACE2, leading to elevated Ang II (Ni et al., 2020). Excess Ang II activates NOX (Wen et al., 2012), a major ROS generator, driving lung injury, hypoxemia (low SpO_2_), and disease progression (Forcados et al., 2021). Ang II also enhances endothelial activation by upregulating adhesion molecules and pro-inflammatory cytokines, fuelling inflammation, thrombosis, and poor outcomes (Dandona et al., 2007).

### 3.3 Oxidative stress, inflammation, and COVID-19

In COVID-19, OS acts as a central driver of inflammation. Excess ROS from NOXs, mitochondrial dysfunction, and uncoupled endothelial nitric oxide NO synthase activate redox-sensitive factors (NF-κB, NF-κB and activator protein-1 (AP-1)), inducing cytokines (IL-6, IL-1β, TNF-α). OS also triggers NLRP3 inflammasome activation, promoting IL-1β and IL-18 release, thus fueling the cytokine storm in severe disease (Smail et al., 2021; Wang et al., 2025). ROS generate oxidative modifications of lipids and proteins, including malondialdehyde (MDA) adducts and nitrotyrosine residues, which act as DAMPs. These are sensed by PRRs such as Toll-like receptors (TLRs), activating innate immune cells and amplifying inflammation. This feed-forward loop explains why elevated oxidative biomarkers (MDA, 8-isoprostanes, nitrotyrosine) correlate with systemic inflammation and progression to ARDS in COVID-19 (Pavlova et al., 2025). OS aggravates endothelial dysfunction by reducing NO bioavailability and increasing peroxynitrite (ONOO^−^) formation. This activates adhesion molecules (ICAM-1, VCAM-1), driving leukocyte infiltration, microvascular injury, thrombosis, and multi-organ damage. Together, these processes establish a pathogenic OS–inflammation cycle that underlies COVID-19 severity and poor prognosis (Jin et al., 2024).

## 4 The promise of oxidative prognostic biomarkers in COVID-19

A prognostic biomarker is a measurable biological indicator that predicts disease course, severity, or outcome independent of treatment. In COVID-19, prognostic biomarkers help identify patients at risk of severe illness, ICU admission, or mortality. Key properties include sensitivity, specificity, reproducibility, and stability across biological matrices, enabling early detection of systemic inflammation or OS (Cagney et al., 2018). Prognostic biomarkers enable risk stratification, resource optimization, and timely interventions to reduce complications such as ARDS and multi-organ failure. OS biomarkers (e.g., MDA, 8-isoprostanes [8-iso-PGF_2_α]) together with antioxidant enzyme levels have been associated with disease severity and adverse outcomes, underscoring their value in clinical decision-making (Wang et al., 2025).

Early studies during the pandemic highlighted MDA, 8-iso-PGF_2_α, and nitrotyrosine as prognostic indicators of COVID-19, reflecting lipid peroxidation and oxidative damage to proteins and nucleic acids. Elevated MDA levels, in particular, have been linked to critical disease, underscoring the contribution of OS to adverse outcomes such as ARDS (Smail et al., 2023). Studies assessing panels of OS biomarkers (e.g., thiobarbituric acid reactive substances (TBARS) as a proxy for MDA) alongside antioxidant defences (SOD, CAT, GR) revealed a pronounced imbalance in COVID-19 patients (Pavlova et al., 2025). These findings support the prognostic promise of oxidative biomarkers for predicting disease severity and potential progression toward respiratory failure. The behaviour of antioxidant enzymes highlights their prognostic value. In COVID-19 patients, SOD activity was markedly upregulated (∼5-fold) as a compensatory response to ROS. CAT activity also increased (∼1.4–1.5-fold), whereas GR activity declined (∼2.5-fold), reflecting a pattern of early enzymatic activation followed by exhaustion with disease progression (Pavlova et al., 2025). Cofactor micronutrients for endogenous antioxidants including Zn, Se, Cu, and Mn have been identified as dual prognostic markers and therapeutic targets in COVID-19. Epidemiological studies associate deficiencies in these minerals with impaired antioxidant enzyme activity and poorer clinical outcomes (Al-Fartusie et al., 2023; Dresen et al., 2023; Smail et al., 2023).

### 4.1 Serum antioxidant enzymes and their genetic polymorphisms, as biomarkers of COVID-19 severity and mortality

**FIGURE 4:**
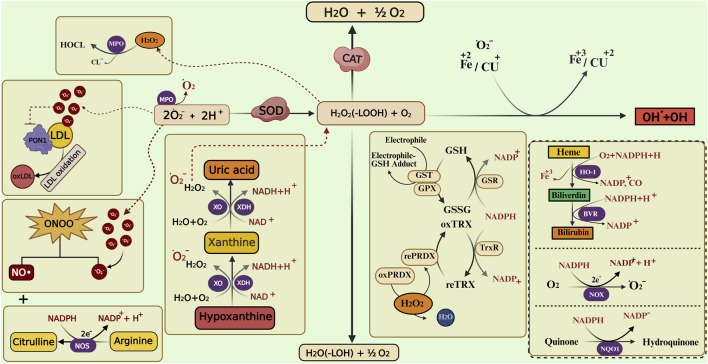
Enzymatic and non-enzymatic pathways involved in reactive oxygen and nitrogen species metabolism. The figure illustrates the interplay between different antioxidant enzymes (such as SOD, CAT, GPX, GST, PRDX, and HO-1) and non-enzymatic antioxidants (bilirubin, uric acid) in neutralizing reactive oxygen species (ROS) and reactive nitrogen species (RNS). It highlights the conversion of superoxide (O_2_•^-^) to hydrogen peroxide (H_2_O_2_), the role of catalase and glutathione-related enzymes in detoxification, and the involvement of xanthine oxidase (XO), nitric oxide synthase (NOS), and heme oxygenase pathways in redox balance. Transition metals (Fe, Cu) catalyze the Fenton reaction producing hydroxyl radicals (•OH), while lipid peroxidation and peroxynitrite (ONOO^−^) formation contribute to oxidative damage. Abbreviations: SOD, superoxide dismutase; CAT, catalase; MPO, myeloperoxidase; XO, xanthine oxidase; XDH, xanthine dehydrogenase; NOS, nitric oxide synthase; NOX, NADPH oxidase; NQO1, NAD(P)H quinone dehydrogenase 1; GPX, glutathione peroxidase; GST, glutathione S-transferase; GSR, glutathione reductase; PRDX, peroxiredoxin; rePRDX, reduced peroxiredoxin; oxPRDX, oxidized peroxiredoxin; TrxR, thioredoxin reductase; HO-1, heme oxygenase-1; BVR, biliverdin reductase; PON1, paraoxonase 1; LDL, low-density lipoprotein; oxLDL, oxidized low-density lipoprotein; NADPH, nicotinamide adenine dinucleotide phosphate (reduced form); NADP^+^, nicotinamide adenine dinucleotide phosphate (oxidized form); NAD^+^, nicotinamide adenine dinucleotide; O_2_•^-^, superoxide anion; H_2_O_2_, hydrogen peroxide; •OH, hydroxyl radical; ONOO^−^, peroxynitrite. The image was created using the BioRender program, available at https://www.biorender.com.

**FIGURE 5:**
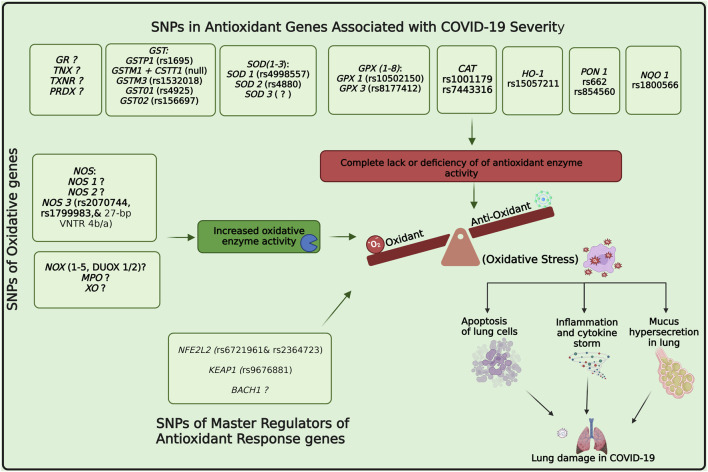
Role of oxidative/antioxidant enzyme activity and genetic variations in COVID-19 severity. SNPs in oxidative and antioxidant genes (GSTs, SODs, GPXs, CAT, HO-1, PON1, NQO1) and master regulators (NFE2L2, KEAP1, BACH1) modulate redox balance during SARS-CoV-2 infection. Imbalances can shift the equilibrium toward oxidative stress, leading to excess oxidant production, apoptosis of lung cells, cytokine storm, and mucus hypersecretion. Red box indicates deficiency or loss of antioxidant enzyme activity, while the green box denotes increased oxidative enzyme activity that enhances radical production. Together, these processes contribute to lung injury and poor outcomes in COVID-19. Abbreviations: GR, glutathione reductase; TNX, thioredoxin; TXNR, thioredoxin reductase; PRDX, peroxiredoxin; GST, glutathione S-transferase; GSTP1, glutathione S-transferase pi 1; GSTM1, glutathione S-transferase mu 1; GSTM3, glutathione S-transferase mu 3; GSTO1, glutathione S-transferase omega 1; GSTO2, glutathione S-transferase omega 2; SOD, superoxide dismutase; GPX, glutathione peroxidase; CAT, catalase; HO-1, heme oxygenase 1; PON1, paraoxonase 1; NQO1, NAD(P)H quinone dehydrogenase 1; NOS, nitric oxide synthase; NOX, NADPH oxidase; DUOX, dual oxidase; MPO, myeloperoxidase; XO, xanthine oxidase; NFE2L2, nuclear factor erythroid 2-related factor 2; KEAP1, Kelch-like ECH-associated protein 1; BACH1, BTB domain and CNC homology 1. The image was created using the BioRender program, available at https://www.biorender.com.

**FIGURE 6:**
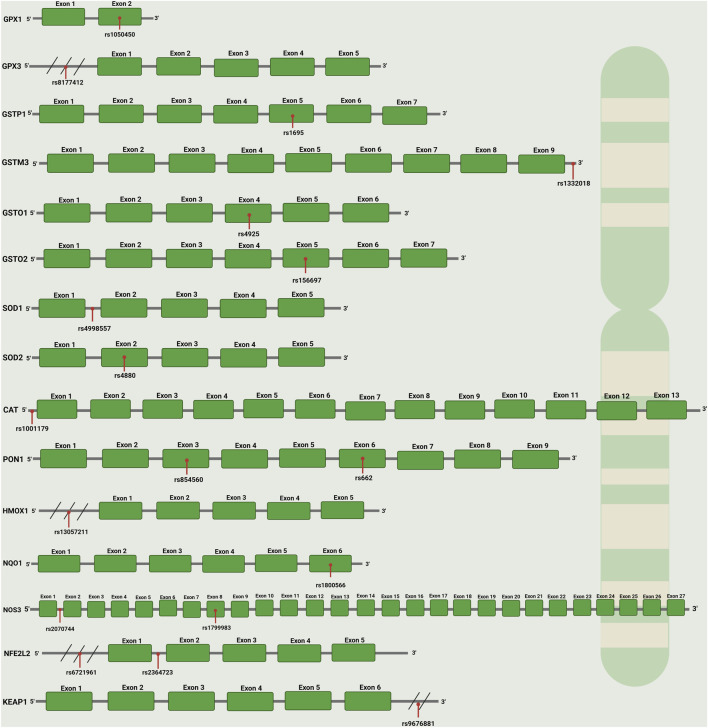
Gene structures, exon–intron organization, and SNP locations of antioxidant/oxidative stress-related genes. The schematic diagram shows the gene structures of GPX1, GPX3, NOS3, and GSTP1, indicating the number of exons (green boxes), introns (connecting lines), and the position of selected SNPs (red bars). Abbreviations: GPX1: Glutathione Peroxidase 1; GPX3: Glutathione Peroxidase 3; GSTP1: Glutathione S-Transferase Pi 1; GSTM3: Glutathione S-Transferase Mu 3; GSTO1: Glutathione S-Transferase Omega 1; GSTO2: Glutathione S-Transferase Omega 2; SOD1: Superoxide Dismutase 1; SOD2: Superoxide Dismutase 2; CAT: Catalase; PON1: Paraoxonase 1; HMOX1: Heme Oxygenase 1; NQO1: NAD(P)H Quinone Dehydrogenase 1; NOS3: Nitric Oxide Synthase three (endothelial NOS); NFE2L2: Nuclear Factor, Erythroid 2-Like 2 (Nrf2); KEAP1: Kelch-Like ECH-Associated Protein 1. SNP: Single Nucleotide Polymorphism. The image was created using the BioRender program, available at https://www.biorender.com.

They make up the first line of antioxidant defense and prevent oxidative damage by directly interacting with ROS. They act as catalysts and are efficiently recycled after working. The enzymes that make up this enzymatic antioxidant system are GPx, GR, glutathione S-transferases (GST), SOD, CAT, paraoxonase 1 (PON1), Heme oxygenase-1 (HO-1), NAD(P)H:quinone oxidoreductase 1 (NQO1), and thioredoxin (TXN)/thioredoxin reductase (TXNRD), and peroxiredoxins (PRDXs) (Boots et al., 2008), their functions were summarised in Figure 4. Table 1 summarizes their serum activity as prognostic biomarkers, whereas Table 2 and Figure 5 present their related SNPs. Finally, Table 3 and Figure 6 present the gene structure, including exon and intron counts, SNP positions, and ClinVar annotations.

**TABLE 1:** Prognostic significance of serum antioxidant and oxidative enzymes in COVID-19.

Marker/System	Definition and function	Measurement	COVID-19 associations	Prognostic value	Reference(s)	Country/Region	Total sample size (N)
GPx activity	Catalyzes reduction of H_2_O_2_ and lipid peroxides using GSH.	ELISA/spectrophotometry	Outpatients ↑ GPx vs. controls (Golabi et al., 2022; Ignacio-Mejía et al., 2025); severe cases ↓ GPx and GPx3 (reversible w/Se) (28). No significant serum changes (Smail et al., 2023)	Elevated = compensatory; reduced in severe = overwhelmed redox	Golabi et al. (2022), Ignacio-Mejía et al. (2025), Smail et al. (2023)	Mexico, Iran, Iraq	54 (54 post-COVID +0 controls); 106 (53 COVID +53 controls); 180 (120 COVID +60 controls)
GR activity	Regenerates GSH from GSSG via NADPH.	Enzymatic assays	Low GR + high GPx + low thiols distinguish severe	Reduced GR correlates with severity and pulmonary involvement	Wolszczak-Biedrzycka et al. (2024a)	Poland	335 (218 COVID +69 post-COVID +48 controls)
SOD activity	Converts O_2_^−^• → H_2_O_2_	Serum enzyme assays	Elevated in ICU vs. non-ICU (Wolszczak-Biedrzycka et al., 2024a). Decreased in Omicron-infected; correlates w/severity (Chu et al., 2024). Lower in COVID vs. controls (Smail et al., 2023)	Reflects oxidative burden; ↑ in severe, ↓ in chronic	(Wolszczak-Biedrzycka et al., 2024a; Chu et al., 2024) Smail et al. (2023)	Poland, China, Iraq	335 (218 COVID +69 post-COVID +48 controls); 139 (109 COVID +30 controls); 180 (120 COVID +60 controls)
CAT activity	Decomposes H_2_O_2_ → H_2_O+ O_2_	Enzyme assays	Increased in COVID (Mehri et al., 2021). No significant changes in some studies (Wolszczak-Biedrzycka et al., 2024a; Smail et al., 2023)	Mixed; early response potential but inconsistent prognostic value	(Mehri et al., 2021; Wolszczak-Biedrzycka et al., 2024a) Smail et al. (2023)	Iran, Poland, Iraq	48 (24 COVID +24 controls); 335 (218 COVID +69 post-COVID +48 controls); 180 (120 COVID +60 controls)
HO-1 activity	Degrades heme → biliverdin, CO, Fe; anti-inflammatory/antioxidant	ELISA	Levels ↑ with severity; correlate w/LDH, CRP, CT; AUC ICU prediction 0.816 (Hara et al., 2022) . Also poor prognostic marker in sepsis/mortality (Chen et al., 2022)	Strong severity, ICU, mortality marker	(Hara et al., 2022) Chen et al. (2022)	Japan; Taiwan	83 (64 COVID +19 controls); 256 (156 COVID +100 controls)
PON1 activity	HDL esterase preventing lipid peroxidation	Arylesterase assay	↓ in COVID vs. controls; high diagnostic ROC accuracy. Not severity- or mortality-linked	Diagnostic use only	Barrios et al. (2022)	Spain	1338 (615 COVID +50 controls)
Other enzymes (GST, TXN, TXNR, NQO1, PRDXs)	Detoxification, redox regulation, cytoprotection	Biochemical/genetic assays	Limited or no serum data in COVID-19	Not supported clinically (insufficient evidence)	N/A	N/A	N/A
NOX activity	Generates ROS (O_2_^−^•) from NADPH.	Indirect (ROS/gene expression)	SARS-CoV-2 → NOX2 activation → ↑ ROS and inflammatory mediators, incl. CNS microglia	Mechanistic only; no serum prognostic use	Sindona et al. (2021)	Italy (Review)	N/A
XO activity	Produces ROS (O_2_^−^•, H_2_O_2_) in purine metabolism	Metabolic profiling	↑ Xanthine in severe COVID-19	Therapeutic target; prognostic metabolite	Xu et al. (2023)	N/A (Review)	N/A
MPO activity	Produces HOCl via neutrophil response	Serum enzyme/protein assays	Elevated in mild vs. controls; not different in hospitalized (Xu et al., 2023)	Limited prognostic use; early oxidative burst	Xu et al. (2023)	N/A (Review)	N/A
NOS activity	Generates NO, modulating vascular/immune responses	Serum nitrate/nitrite levels	Higher nitrate levels predict 30-day mortality, independent of severity (Smail et al., 2023).	Strong mortality predictor	Smail et al. (2023)	Iraq	180 (120 COVID +60 controls)

Abbreviations: GPx, glutathione peroxidase; GSH, reduced glutathione; GR, glutathione reductase; GSSG, oxidized glutathione; NADPH, nicotinamide adenine dinucleotide phosphate (reduced form); SOD, superoxide dismutase; O_2_^−^•, superoxide anion; H_2_O_2_, hydrogen peroxide; CAT, catalase; HO-1, heme oxygenase-1; LDH, lactate dehydrogenase; CRP, C-reactive protein; CT, computed tomography; PON1, paraoxonase-1; HDL, high-density lipoprotein; GST, glutathione S-transferase; TXN, thioredoxin; TXNR, thioredoxin reductase; NQO1, NAD(P)H quinone oxidoreductase-1; NOX, NADPH, oxidase; XO, xanthine oxidase; MPO, myeloperoxidase; HOCl, hypochlorous acid; NOS, nitric oxide synthase; NO, nitric oxide; PRDXs, peroxiredoxins; ROC, receiver operating characteristic; AUC, area under the curve.

**TABLE 2:** SNPs in oxidative and antioxidative genes linked to COVID-19.

Gene symbol	Notable SNP(s)	Study (ies) (Country, total sample size, COVID-19, healthy controls)
1. SNP (s) of Antioxidative Genes		
*GPX1*	rs1050450 (Pro200Leu)	Although the GPX1 rs1050450 polymorphism was not associated with COVID-19 susceptibility, it was significantly linked to increased D-dimer levels (p = 0.009) in Leu/Leu homozygous COVID-19 patients, and showed a borderline association with elevated fibrinogen (Jerotic et al., 2022) (Serbia, 458, 229, 229)
*GPX3*	rs8177412	There was a statistically significant link between the GPX3 rs8177412 variant genotype and an increased risk of developing severe COVID-19, with an odds ratio of 2.42 (p = 0.032) (Markovic et al., 2023) (Serbia, 265, 265, N/A)
*GR*	rs2257151	No study has yet established an association with COVID-19 severity
*GSTP1*	rs1695 (Ile105Val)	There was no meaningful association between the rs1695 (Ile105Val) polymorphism and the likelihood of developing severe COVID-19 (Markovic et al., 2023) (Serbia, 265, 265, N/A)
*GSTM1 and GSTT1*	Null genotype	Although the GSTM1^−/−^ and GSTT1^−/−^ genotypes were more frequent among patients with severe COVID-19, this difference did not reach statistical significance (p > 0.05), indicating no clear association with disease severity. However, the GSTT1^−/−^ genotype was significantly linked to increased mortality risk, with a hazard ratio of 2.28 (p = 0.047), and the combined genotype GSTM1^+/+^ with GSTT1^−/−^ showed an even higher risk of death (HR = 2.72, p = 0.02) (Abbas et al., 2021) (India, 269, 269, N/A)
*GSTM3*	rs1332018	Despite its role in redox signaling, the GSTM3 rs1332018 variant was not linked to increased risk for severe COVID-19. This indicates its influence, if any, on disease progression is likely minimal or context-dependent (Markovic et al., 2023) (Serbia, 265, 265, N/A)
*GSTO1*	rs4925	GSTO1 rs4925 did not show any meaningful association with the likelihood of experiencing severe forms of COVID-19 (Markovic et al., 2023)
*GSTO2*	rs156697	The GSTO2 rs156697 variant was not significantly linked to an increased risk of developing severe COVID-19 (Markovic et al., 2023) (Serbia, 265, 265, N/A)
*SOD1*	rs4998557	These findings suggest that the SOD1 rs4998557 variant is not associated with susceptibility to COVID-19 in the studied population (Eid et al., 2024) (Russia, 169, 169 (post-COVID), N/A)
*SOD2*	rs4880 (Val16Ala)	1. TT genotype (Val/Val) associated with higher severity (OR 4.34; p = 0.002) (Eid et al., 2024) (Russia, 169, 169 (post-COVID), N/A) 2. The Val/Val (TT) genotype at rs4880 is associated with reduced mitochondrial translocation of SOD2 and up to 30%–40% lower enzymatic activity, potentially resulting in impaired detoxification of O_2_^−•^radicals and elevated oxidative stress (Dakó et al., 2025) (Romania, 110, 59 S-ECC patients, 51) 3. SOD2 rs4880 polymorphism (especially AG and GG variants) may increase oxidative stress, leading to greater susceptibility and severity of caries in children (Dakó et al., 2025) (Romania, 110, 59 S-ECC patients, 51) 4. The SOD2 rs4880 polymorphism did not significantly affect COVID-19 susceptibility. However, the Val/Val genotype was associated with higher levels of fibrinogen and ferritin—two key biomarkers of inflammation and coagulation in COVID-19 patients (p = 0.040 and 0.033, respectively) (Jerotic et al., 2022) (Serbia, 458, 229, 229)
*CAT*	rs1001179 (C262T)	the comparison of genotype frequencies between patients and controls also yielded no significant difference (p = 0.72), and the allele frequencies were likewise not statistically different (p = 0.88) (Eid et al., 2024) (Russia, 169, 169 (post-COVID), N/A)
*PON1*	rs662 (Q192R)	1. The Ghoreshi et al. (2022)cohort confirms the G (R) allele’s association with disease severity and reduced survival, along with lower enzyme activity in acute illness (Ghoreshi et al., 2022) (Iran, 470, 470, N/A) 2. The Arslan study (2025) suggests a distinct shift in genotype frequencies among severe COVID-19 patients—particularly an elevated QR genotype and decreased RR genotype (Arslan et al., 2025) (Turkey, 50, 26, 24) 3. In Saadat (2022), the PON1 Q192R (rs662) polymorphism showed no significant association with COVID-19 prevalence or mortality after adjusting for confounders (Saadat, 2022) (N/A, N/A, N/A)
*HMOX1 (H O -1)*	rs13057211	A novel SNP (A > G, rs13057211) located within the GT(n) region has recently been identified in COVID-19 patients. This variant was significantly associated with higher mortality (e.g., OR ≈ 3.7, p = 0.021), whereas the length of GT repeats alone did not correlate with survival or HO-1 serum levels (Fares et al., 2023) (Egypt, 90, 90, N/A)
*NQO1*	rs1800566 (C609T)	The T allele (Pro187Ser) produces a less stable enzyme with reduced activity. This SNP has been tentatively associated with poor prognosis and increased inflammatory biomarkers in COVID-19 patients, though more validation is needed
*TXN,/TXNR, and PRDXs*	Many SNPs	No available research has reported an association with COVID-19 severity
2. SNP (s) of Oxidative Genes		
*NOX, XO, MPO*	Many SNPs	No studies have so far demonstrated a relationship with COVID-19 severity
*NOS3*	rs1799983 (Glu298Asp or 894G>T)	An analysis of 180 Turkish patients (December 2021–September 2022) evaluated NOS3 rs1799983. No statistically significant difference was found between mild and severe disease groups overall (GG, GT, TT distributions were similar) (İdikut et al., 2025) (Turkey, 180, 180, N/A)
	rs2070744 (T–786C)	Homozygous AA genotype of PON1 rs662 and TT genotype of NOS3 rs2070744 were found to be significantly more frequent among patients with severe COVID-19, indicating a potential genetic predisposition to worse outcomes. In contrast, individuals carrying the heterozygous AG genotype of PON1 and TC genotype of NOS3 showed a significantly higher prevalence in the mild COVID-19 group, suggesting that these genotypes may confer a protective effect against severe disease progression in Russia (Eid et al., 2023) (N/A, N/A, N/A)
	27-bp VNTR 4b/a)	27-bp VNTR 4b/a) in 180 COVID-19 patients and found no statistically significant associations with disease severity overall. However, in a subgroup of patients under 50 years old without COPD, there was a trend toward increased frequency of the 4b/4b genotype and 4b allele among severe cases, suggesting a possible role of NOS3 genetic variants in younger individuals’ susceptibility to severe COVID-19 (İdikut et al., 2025) (Turkey, 180, 180, N/A)
3. Master Regulators of Antioxidant Response and Their Genetic Polymorphism		
*NFE2L2*	rs6721961	Despite Nrf2’s central regulatory role in oxidative stress responses, the rs6721961 promoter SNP did not significantly influence COVID-19 risk or disease biomarkers. This suggests that reduced Nrf2 transcriptional activity alone may not critically affect the host response to SARS-CoV-2 infection (Markovic et al., 2023) (Serbia, 265, 265, N/A)
	rs2364723	The study identified that the NFE2L2 (NRF2) rs2364723 G allele plays a protective role against severe COVID-19 pneumonia, particularly under the recessive model (GG genotype), suggesting enhanced antioxidant defense through the NRF2-KEAP1 pathway (Soto et al., 2022) (Mexico, 221, 110, 111)
*KEAP1*	rs9676881	KEAP1 SNPs rs9676881 G allele and rs34197572 T allele were significantly associated with more aggressive COVID-19 outcomes, likely due to compromised redox regulation and disrupted transcriptional control of antioxidant responses (Soto et al., 2022) (Mexico, 221, 110, 111)
*BACH1*	Many SNPs	Thus far, no evidence has been published connecting it with COVID-19 severity

Abbreviations: GPX1/GPX3, glutathione peroxidase-1/3; GR, glutathione reductase; GSTP1/GSTM1/GSTM3/GSTO1/GSTO2, glutathione S-transferase isoforms; SOD1/SOD2, superoxide dismutase-1/2; CAT, catalase; PON1, paraoxonase-1; HMOX1 (HO-1), heme oxygenase-1; NQO1, NAD(P)H quinone oxidoreductase-1; TXN, thioredoxin; TXNR, thioredoxin reductase; PRDXs, peroxiredoxins; NOX, NADPH, oxidase; XO, xanthine oxidase; MPO, myeloperoxidase; NOS3, endothelial nitric oxide synthase; NFE2L2 (NRF2), nuclear factor erythroid 2-related factor 2; KEAP1, Kelch-like ECH-associated protein 1; BACH1, BTB, domain and CNC, homolog 1; VNTR, variable number tandem repeat; COPD, chronic obstructive pulmonary disease; OR, odds ratio; HR, hazard ratio; SNP, single nucleotide polymorphism. Polymorphisms are indicated by dbSNP IDs (rs numbers) and, where applicable, amino acid substitutions. Reported associations reflect study-specific findings, with non-significant results indicating no clear correlation in the studied cohorts.

**TABLE 3:** ClinVar-reported significance and position of SNPs in oxidative and antioxidative genes.

Gene	SNP	Chromosome position	Location in gene	Alleles	Exons gene number	Introns gene number	Reported in ClinVar
GPX1	rs1050450	chr3:49357401	Exon 2	G>A/G>C/G>T	2	1	Benign
GPX3	rs8177412	chr5:151020526	Upstream Transcript Variant	T>C	5	4	Not reported
GSTP1	rs1695	chr11:67585218	Exon 5	A>G/A>T	7	6	Benign
GSTM3	rs1332018	chr1:109740350	5′ UTR variant	G>A/G>T	9	8	Not reported
GSTO1	rs4925	chr10:104263031	Exon 4	C>A	6	5	benign
GSTO2	rs156697	chr10:104279427	Exon 5	A>C/A>G/A>T	7	6	Not reported
SOD1	rs4998557	chr21:31662579	Intron 1	G>A	5	4	Not reported
SOD2	rs4880	chr6:159692840	Exon 2	A>C/A>G/A>T	5	4	Conflicting classifications of pathogenicity; risk factor Likely risk allele (1); Benign (1)
CAT	rs1001179	chr11:34464164	Upstream Transcript Variant	C>G/C>T	13	12	Uncertain Significance (VUS)
PON1	rs662	chr7:95308134	Exon 6	T>A/T>C/T>G	9	8	Benign
	rs854560	chr7:95316772	Exon 3	A>C/A>G/A > N/A>T			Benign
HMOX1	rs13057211	chr22:35380865	Upstream Transcript Variant	A>C/A>G/A>T	5	4	Not reported
NQO1	rs1800566	chr16:69711242	Exon 6	G>A/G>C/G>T	6	5	Uncertain significance
NOS3	rs1799983	chr7:150999023	Exon 8	T>A/T>G	27	26	Benign/risk factor
	rs2070744	chr7:150992991	Intron 1	C>G/C>T			Protective/risk factor
NFE2L2	rs6721961	chr2:177265309	Upstream Transcript Variant	T>A/T>C/T>G	5	4	Not Reported in ClinVar
	rs2364723	chr2:177261818	Intron 1	G>A/G>C/G>T			Not reported
KEAP1	rs9676881	chr19:10486104	Downstream Transcript Variant	G>A/G>C/G>T	6	5	Not reported

Clinical significance was derived from ClinVar when available. Classifications include: Benign, Likely Benign, VUS (Variant of Uncertain Significance), Likely Pathogenic, and P Pathogenic. Variants not listed in ClinVar are indicated as Not reported. All informations were derived from NCBI. Abbreviations: GPX1/3, glutathione peroxidase-1/3; GSTP1/GSTM3/GSTO1/GSTO2, glutathione S-transferase isoforms; SOD1/2, superoxide dismutase-1/2; CAT, catalase; PON1, paraoxonase-1; HMOX1 (HO-1), heme oxygenase-1; NQO1, NAD(P)H quinone oxidoreductase-1; NOS3, endothelial nitric oxide synthase-3; NFE2L2 (NRF2), nuclear factor erythroid 2-related factor 2; KEAP1, Kelch-like ECH-associated protein 1; SNP, single nucleotide polymorphism; UTR, untranslated region.

#### 4.1.1 Glutathione peroxidase and glutathione reductase

Cells rely on glutathione (GSH), a tripeptide composed of glutamate, cysteine, and glycine, as a central antioxidant and redox regulator. In its reduced form, GSH donates electrons to neutralize ROS, including H_2_O_2_ and lipid peroxides, being oxidized to glutathione disulfide (GSSG) in the process (Figure 4). Indeed, the ratio of GSH to GSSG serves as a sensitive measure of OS within cells: a high GSH/GSSG ratio signals healthy redox balance, whereas a shift toward GSSG reflects redox disturbance and potential cellular damage (Diaz-Del Cerro et al., 2023).

GPxs are a family of eight enzymes (GPx1–GPx8) that leverages this redox power of GSH. GPx reduces H_2_O_2_ to water (or lipid hydroperoxides to their corresponding alcohols) by utilizing two GSH molecules, converting them into GSSG (Figure 4). At the molecular level, this occurs through the oxidation of a selenocysteine residue in GPx, followed by sequential interactions with GSH that regenerate the enzyme’s active form and release oxidized GSH Most GPx isoforms, including GPx1 to GPx3, function as tetramers composed of four identical subunits, each with a molecular weight of approximately 22–23 kDa [105]. GPx4 is unique in that it functions as a monomer. GPx1 and GPx4 are widely distributed across cellular compartments such as the cytosol, mitochondria, and nucleus, and are expressed in a broad range of cell types throughout the body, supporting general cellular protection against OS (Smeyne and Smeyne, 2013). The study by Golabi et al. (2022) concluded that serum concentrations of GPx were significantly higher in COVID-19 outpatients compared to non-infected controls, even after adjusting for dietary supplement use. This elevated GPx level suggests that the body enhances its antioxidant defense mechanisms during SARS-CoV-2 infection, possibly as a compensatory response to increased OS.

GPx1 is a cytosolic enzyme that reduces H_2_O_2_ and organic hydroperoxides using GSH, thereby protecting cells from oxidative damage. The rs1050450 polymorphism (Pro198Leu) leads to a Proline-to-Leucine amino acid change, resulting in reduced enzymatic activity of the Leu variant. This reduced activity may impair redox homeostasis, particularly under conditions of OS (Tang et al., 2008; Zhang et al., 2014). GPx3 is the only extracellular GPx and plays a protective role in plasma and interstitial fluids by detoxifying H_2_O_2_ and lipid peroxides. The rs8177412 polymorphism is located in the promoter region of the gene and is known to downregulate gene expression, resulting in reduced plasma GPx3 activity (Bumbasirevic et al., 2022).

GR plays a critical role in cellular defense against OS triggered by elevated levels of ROS. In mammals, GR enzymatic activity is found in both the cytosol and mitochondria [5]. Despite being localized in different cellular compartments, the mitochondrial and cytosolic forms of GR are biochemically identical, indicating that they are likely produced from a single nuclear gene. This gene is located on chromosome 8p21.1, spans approximately 50 kilobases, and contains 13 exons (Kamerbeek et al., 2007). There is currently no published evidence identifying GR gene variants that influence COVID-19 outcomes To sustain the antioxidant cycle, GR reduces GSSG back to two GSH molecules in an NADPH-dependent reaction. This regeneration maintains the high intracellular GSH/GSSG ratio essential for cellular redox homeostasis. The full GSH–GPx–GR cycle thus enables continuous detoxification of peroxides and sustains a robust antioxidant defense system (Trevisan et al., 2014). A 2025 study using machine learning noted that low GR, along with other OS biomarkers (e.g., high GPx, low thiols), was characteristic of more severe COVID-19 cases. This suggests that diminished GR activity may correlate with greater pulmonary involvement (Raspado et al., 2025). Some data indicate that in severe or fatal COVID-19, GR activity may decline, potentially due to OS overwhelming the antioxidant systems. However, these findings come from smaller or less conclusive reports (Wolszczak-Biedrzycka et al., 2024a).

#### 4.1.2 GST family (Glutathione S-Transferases)

Mammalian cytosolic GSTs are a diverse family of phase II detoxification enzymes categorized into seven major classes based on their sequence homology, immunological properties, and substrate specificity. These classes include: Alpha (GSTA), Mu (GSTM), Pi (GSTP), Theta (GSTT), Sigma (GSTS), Zeta (GSTZ), and Omega (GSTO). Each class plays specialized roles in xenobiotic metabolism, redox regulation, and cell signaling, using reduced GSH to detoxify electrophilic compounds and protect against OS (Ramkumar et al., 2016).

Beyond this catalytic role, GSTP1 exerts non-enzymatic regulatory functions: it physically interacts with the c-Jun N-terminal kinase (JNK) to suppress kinase activity and limit apoptosis under non-stress conditions; OS disrupts this complex, enabling JNK activation and downstream signaling. Similarly, GSTM1 interacts with apoptosis signal-regulating kinase 1 (ASK1) to sequester it and block activation of JNK and p38 pathways, thereby integrating GST function tightly with redox-regulated MAPK signaling (Pajaud et al., 2012). The rs1695 single-nucleotide polymorphism (SNP), also known as Ile105Val (A313G), in GSTP1, results in an amino acid substitution at codon 105 from isoleucine (Ile) to valine (Val). This polymorphism leads to structural changes in the enzyme’s active site, altering its substrate specificity and reducing its enzymatic activity (Ali-Osman et al., 1997). The rs1332018 (A-63C) variant is located in the promoter region of GSTM3, and it has been shown to downregulate gene expression. Carriers of the AC or CC genotypes display significantly lower GSTM3 levels.

In a case–control study of 207 COVID-19 patients *versus* 252 matched controls, GSTP1 rs1695 (Ile105Val) variant carriers had significantly lower odds of developing COVID-19 (p = 0.002), while individuals with the GSTM3 CC genotype showed increased susceptibility (p = 0.024) (Coric et al., 2021b). Moreover, combined risk genotypes across GSTP1 and GSTM3 conferred higher risk for both incidence and severity of COVID-19 (p = 0.001 and p = 0.025, respectively) (Coric et al., 2021b). In a cohort of hospitalized Polish patients (vaccinated and unvaccinated), the GSTP1 Ile/Val genotype was associated with a ∼2.75-fold increased odds of severe COVID-19 among previously vaccinated individuals (p = 0.0398), though no significant link was observed in the unvaccinated group (Orlewska et al., 2023).

A cross-country ecological study by Saadat (2020b) investigated the association between the GSTP1 Ile105Val polymorphism (rs1695) and COVID-19 prevalence and outcomes, using data from 45 nations. After adjusting for confounders such as healthcare infrastructure and national income, researchers found that a higher frequency of the Val105 allele was significantly associated with increased COVID-19 prevalence and mortality (but not case fatality). This association was even stronger in high-income countries, suggesting that the Val105 variant—linked to reduced GSTP1 detoxification capacity may contribute to higher OS, thereby increasing susceptibility to and severity of SARS-CoV-2 infection.

Both GSTM1 and GSTT1 genes can exhibit homozygous gene deletions, known as null genotypes, resulting in complete absence of enzyme activity. A study investigating the relationship between GSTM1 and GSTT1 gene polymorphisms and COVID-19 severity and outcomes analyzed 269 RT-PCR-confirmed patients (149 mild, 120 severe cases). Although the frequencies of GSTM1-null, GSTT1-null, and combined GSTM1^−/−^/GSTT1^−/−^ genotypes were higher in severe cases, no statistically significant association with disease severity was observed. However, mortality risk was significantly higher (2.28-fold) in patients carrying the GSTT1^−/−^ genotype (*p* = 0.047), and individuals with the GSTM1^+/+^ and GSTT1^−/−^ combination had the poorest survival rates (*p* = 0.02). These findings suggest that GSTT1-null genotype may be a genetic risk factor for increased mortality in COVID-19 patients due to impaired OS defense mechanisms (Abbas et al., 2021). The GSTT1-null genotype was associated with increased mortality in COVID-19 patients, likely due to impaired ability to mitigate the oxidative burst and cytokine storm associated with severe disease (Labarrere and Kassab, 2022).

In an ecological study by Saadat (2020a) across 67 countries, Saadat (2020b) explored the relationship between GSTT1 and GSTM1 null genotypes and COVID-19 outcomes. After adjusting for confounding variables like healthcare access, income, and testing rates, the analysis found that a higher frequency of the GSTT1-null genotype was unexpectedly associated with lower COVID-19 mortality and fatality rates (*p* = 0.001 and *p* = 0.005), while GSTM1-null genotype showed no significant correlation. This inverse association suggests a possible protective effect of GSTT1 deletion at the population level, potentially due to differences in lung tissue expression and redox response mechanisms. However, the study emphasizes that these findings are hypothesis-generating and should not be interpreted as causal without further individual-level studies.

GSTO1 and GSTO2 are unique members of the omega-class GSTs, distinguished from other GST families by their thioltransferase activity and their use of cysteine, rather than serine or tyrosine, at the active site. This biochemical difference endows them with deglutathionylation capability, enabling them to reverse protein S-glutathionylation—a key regulatory mechanism in redox signaling and cell stress responses (Board, 2011). GSTO1-1 is involved in the activation of pro-inflammatory cytokines such as interleukin-1β (IL-1β) by reducing disulfide bonds in the IL-1β precursor, thereby linking redox control to inflammation (Kim et al., 2017). Additionally, both GSTO1 and GSTO2 exhibit dehydroascorbate reductase activity, helping regenerate vitamin C and maintain antioxidant defenses (Schmuck et al., 2005). In the study by Markovic et al. (2023), the glutathione-related polymorphisms *GSTO1 rs4925* and *GSTO2 rs156697*, previously linked to increased susceptibility to COVID-19, were not found to significantly influence the severity of the disease. Analysis of 265 hospitalized patients showed no association between these variant genotypes and progression to severe COVID-19 after adjusting for inflammatory biomarkers and clinical covariates (OR = 1.33, *p* = 0.427 for *GSTO1*; OR = 1.05, *p* = 0.892 for *GSTO2*). While these genes encode enzymes involved in redox regulation through GSH metabolism and deglutathionylation—key processes in modulating OS—their polymorphisms appear to affect infection susceptibility rather than clinical progression.

#### 4.1.3 Superoxide dismutase enzyme

It catalyzes the conversion of the O_2_^−•^ to H_2_O_2_. This enzyme is one of the most important cellular defenses against the O_2_^−•^ (Figure 4). Three iso-enzymes are known, each with a specific location and cofactor: SOD_1_ (Cu/Zn-SOD intracellular), locates in the cytoplasm and, to a lesser extent, in the inter-membrane space of the mitochondria; SOD_2_ (Mn-SOD) is in the mitochondrial matrix; and SOD_3_ (Cu/Zn-extracellular SOD), which is analogous to SOD1, but it locates in the extracellular space (Nozik-Grayck et al., 2005). Serum SOD may be used as a biomarker for the prognosis of COVID-19, Mehri et al. (2021) found that the intensive care unit (ICU) COVID-19 patients exhibited higher SOD than non-ICU and healthy controls (HCs). In contrast, the decrease in SOD activity was seen in the serum of COVID-19 compared to HCs (Basaran et al., 2023) (Table 1).

The SNP rs 4998557 is a polymorphism within the SOD1 gene, According to GTEx (the Genotype-Tissue Expression project), rs 4998557 is associated with expression quantitative trait loci (eQTL) for SOD1, suggesting that this variant may influence the gene’s expression levels. The rs4880 polymorphism in SOD2 results in a Val to Alanine (Ala) substitution, which affects mitochondrial import efficiency. The Val/Val genotype has been shown to decrease enzyme transport and activity, contributing to greater accumulation of superoxide anions and a heightened pro-inflammatory state. The SOD1 polymorphism rs4998557 alone is not significantly associated with COVID-19 severity, as shown in genotype and allele frequency analyses. However, when analyzed in combination with SOD2 rs4880 and CAT rs1001179, this SNP contributes to a significant three-locus interaction, increasing the risk of severe COVID-19 (OR ≈ 3.81, *p* ≈ 0.000055). Specifically, carriers of the G allele of rs4998557, along with risk alleles in the other two genes, had nearly threefold increased odds of severe disease. These findings suggest that while rs4998557 alone may not predict COVID-19 outcomes, it plays a modulatory role within OS-related genetic networks, aligning with broader evidence that decreased SOD activity is linked to worse COVID-19 prognosis (Eid et al., 2024). Currently, there’s no published evidence linking SOD3 gene polymorphisms (such as rs1799895, rs2536512, or others) to COVID-19 susceptibility or severity. While SOD3 variants have been implicated in lung function and susceptibility to chronic respiratory conditions (e.g., COPD) (Ganguly et al., 2009), and SOD3 is anatomically and functionally relevant to lungs (NCBI, 2024), no studies to date have reported associations between SOD3 SNPs and COVID-19 outcomes.

#### 4.1.4 Catalase enzyme

The CAT enzyme is a 4-subunits hemoprotein found in a higher concentration in peroxisomes rather than mitochondria. Its primary function is converting H_2_O_2_, produced by beta-oxidation of fatty acids in peroxisomes, into H_2_O and O_2_ (Qin et al., 2020). CAT concentrations and their activity vary depending on the organ, being high in liver tissue and erythrocytes, moderately high in kidney tissue and adipocytes, and low in heart tissue and brain tissue (Deisseroth and Dounce, 1970; Nozik-Grayck et al., 2005). The study conducted by Mehri et al. (2021) suggested that measuring the level of serum CAT activity may predict the prognosis of COVID-19, and it revealed that ICU patients and non-ICU patients have higher CAT activity than the HC group. In contrast, Basaran et al. (2023) documented a non-significant change in the level of serum CAT activity between COVID-19 and HCs.

The rs1001179 variant (also known as C-262T) resides in the promoter region of the CAT gene (−262 from the transcription start site in the 5′-UTR), where it affects transcription factor binding and subsequently alters CAT expression levels (Liu et al., 2016). The T allele has generally been linked to lower catalase enzyme activity and reduced blood CAT levels compared to the C allele, though some studies report conflicting results depending on population and tissue context (Wang et al., 2016). A study by Eid et al. (2024) examining antioxidant enzyme gene variants—including CAT rs1001179—found no significant association between this SNP and COVID-19 severity in their patient cohort (p = 0.72 for genotypes, and p = 0.88 for allele frequencies). Additionally, CAT enzymatic activity did not differ significantly between mild *versus* severe cases, aligning with the genetic findings but the same study uncovered a significant three-locus interaction involving SOD1, SOD2, and CAT rs1001179. Specifically, individuals with the SOD1 G, SOD2 T, and CAT C combination had higher odds of severe COVID-19 (p = 0.0045; OR = 2.84), suggesting a collective, rather than isolated, influence of these antioxidant genes.

#### 4.1.5 Paraoxonase 1

Paraoxonase 1 (PON1) is a calcium-dependent esterase primarily associated with high-density lipoprotein (HDL), where it plays a crucial role in reducing OS by hydrolyzing lipid peroxides and preventing low-density lipoprotein (LDL) oxidation (Figure 4). This antioxidant activity protects cells and vascular tissues from oxidative damage, contributing to anti-inflammatory and cardioprotective effects. PON1’s ability to degrade oxidized lipids and specific toxins like organophosphates supports its protective role against systemic OS, particularly during infections such as COVID-19, where oxidative imbalance is a key pathological feature. A large study of 615 COVID-19 patients showed that serum PON1 arylesterase activity was significantly decreased in those with COVID-19 compared to HCs (PON1 activity ∼120 U/L vs. ∼213 U/L; p < 0.001). However, there was no difference in PON1 activity based on disease severity or mortality, indicating that while it may help diagnose infection, it does not prognosticate outcomes. The current evidence indicates that serum Paraoxonase-1 (PON1) levels, particularly arylesterase activity, are better suited as a diagnostic biomarker for COVID-19 rather than a prognostic biomarker (Barrios et al., 2022).

Notably, genetic polymorphisms like Q192R (rs662) influence PON1 activity, with the R allele often linked to lower detoxifying efficiency and heightened OS in disease states (Ghoreshi et al., 2022). Saadat (2022) investigated whether common functional polymorphisms of the antioxidant enzyme PON1—Q192R (rs662) and L55M (rs854560)—are associated with global COVID-19 burden. Using prevalence, mortality, and diagnostic testing data from 48 countries (as of 25 November 2020) and adjusting for the Human Development Index and testing rates, the study found that the M55 allele frequency showed a significant positive correlation with both COVID-19 prevalence (partial r = 0.487, p = 0.002) and mortality (partial r = 0.551, p < 0.001), whereas Q192R was not associated (Saadat, 2022).

#### 4.1.6 Heme oxygenase-1

Heme oxygenase-1 (HO-1) is a crucial antioxidant and cytoprotective enzyme that degrades free heme into biliverdin, carbon monoxide (CO), and iron—metabolites that collectively reduce OS and inflammation (Figure 4). Biliverdin (and its product bilirubin) acts as a potent antioxidant, while CO exerts anti-inflammatory and vasodilatory effects. Through these mechanisms, HO-1 modulates immune responses, suppresses ROS, and protects endothelial integrity during systemic inflammation and infection. Hara et al., 2022 conducted a study on 64 COVID-19 patients categorized as mild (n = 11), moderate (n = 38), and severe (n = 15). They found that serum HO-1 levels increased stepwise with disease severity, from approximately 11.0–24.3–59.6 ng/mL across groups (Hara et al., 2022). HO-1 also correlated significantly with biomarkers of lung and systemic injury including lactate dehydrogenase (R = 0.422), C-reactive protein (R = 0.463), and CT imaging scores for ground glass opacity and consolidation (R = 0.625). Importantly, HO-1 outperformed another biomarker (sCD163) in predicting ICU admission (AUC: 0.816 vs. 0.743) and even performed better when combined with imaging and sex in composite models (AUCs around 0.92) groups (Hara et al., 2022; Chen et al., 2022 investigated HO-1 levels in 156 moderate-to-critical COVID-19 patients, assessing its association with early development of sepsis (within 48 h of admission). HO-1 levels were significantly higher in patients who developed sepsis, even after adjustment for confounders. However, no direct link to overall COVID-19 mortality was found (Chen et al., 2022).

A recent study by Fares et al. (2023) identified a novel SNP, rs13057211 (A>G), located in the HMOX1 promoter region, which was significantly associated with increased COVID-19 mortality in two independent patient cohorts. While the commonly studied GT(n) repeat length in the HMOX1 promoter did not correlate with outcomes, carriers of the G allele at rs13057211 had significantly higher odds of mortality (OR = 3.7, *p* = 0.021), potentially due to impaired HO-1 inducibility and reduced protection against infection-induced OS. These findings underscore the critical role of HO-1 in host defense and suggest that rs13057211 may serve as a predictive biomarker for severe COVID-19 outcomes (Fares et al., 2023).

Serum HO-1 has been measured in patients with ARDS and interstitial lung disease exacerbations, demonstrating potential prognostic value in clinical settings (Nagasawa et al., 2020). Similarly, HO-1 has been evaluated in COVID-19 cohorts, where elevated levels correlated with disease severity and outcome (Hara et al., 2022). These examples illustrate the translational feasibility of HO-1 as a prognostic biomarker.

#### 4.1.7 NAD(P)H:quinone oidoreductase 1 (NQO1)

The enzyme NQO1 plays a critical protective role in mitigating OS and inflammation during viral infections, including COVID-19. NQO1 catalyzes the two-electron reduction of quinones to hydroquinones, thereby preventing redox cycling and subsequent ROS generation. This antioxidant defense is particularly important in the context of NOX-mediated ROS production, which has been implicated in the pathogenesis and severity of SARS-CoV-2 infection (Damiano et al., 2020). There was not any any studies showing that NQO1 levels in serum are associated with COVID-19 mortality or disease severity. There is emerging evidence, however, that NQO1 expression may be altered post–COVID-19 in the context of neuroinflammatory processes, but this relates to post-infection neurological effects rather than serving as a prognostic blood biomarker during acute COVID-19 (Yang et al., 2021; Hamdy et al., 2022).

The NQO1 gene is regulated by the Nrf2–Keap1/ARE pathway, which is activated in response to OS induced by viral replication and inflammatory cytokines. Notably, polymorphisms in NQO1, such as C609T (rs1800566) and 465 (C>T) in the human cDNA sequence, lead to reduced or absent enzyme activity, compromising cellular antioxidant capacity and potentially exacerbating the cytokine storm and lung injury (Atia et al., 2014) observed in severe COVID-19 cases.

The NQO1 and NOX enzymes represent a redox axis where NOX enzymes generate ROS, while NQO1 acts to detoxify and protect against ROS-induced damage. In viral infections such as COVID-19, dysregulation of either system—overactive NOX or underactive NQO1 (due to genetic polymorphisms)—can lead to excessive inflammation and tissue injury. Balancing this axis is critical for controlling disease severity.

#### 4.1.8 Thioredoxin/thioredoxin reductase

This is a Core redox pair that keeps protein thiols reduced. TXN donates electrons to peroxiredoxins to remove ROS/RNS, while TXNRD (a selenoprotein) regenerates reduced TXN using NADPH (Figure 4). The TXN system is repeatedly highlighted as a frontline antioxidant/immune-modulating pathway and a druggable node (e.g., auranofin inhibits TXNRD) (Sudhadevi and Harijith, 2024). In a hospital cohort (Croatia; n = 88) sampled at admission, plasma TXN protein and TXNRD activity were significantly elevated in COVID-19 vs. HCs, with the paper’s figures showing system “intensification” most pronounced in those who died within a week; the same study links stronger OS footprints (lipid peroxidation, protein carbonyls) to non-survivors. While not offered as a standalone clinical test, these data support prognostic relevance of an activated TXN/TXNRD axis in severe/fatal disease (Žarković et al., 2022).

To date, no reproducible associations between TXN/TXNRD germline variants and COVID-19 severity have been established. Broader host-genetic reviews/catalogs do not report TXN/TXNRD among validated loci, and recent selenoprotein-polymorphism overviews note that TXNRD variants remain understudied in human disease genetics generally.

#### 4.1.9 Peroxiredoxins

Peroxiredoxins (PRDX), a family (PRDX1-6) of thiol-dependent peroxidases that detoxify H_2_O_2_ and peroxynitrite and relay redox signals. They are physiologically coupled to the TXN/TXNRD system (TXN reduces oxidized PRDX) (Figure 4). PRDXs are widely expressed in lung and immune cells and are active in viral infections (Karpenko et al., 2021).

Direct adult-COVID serum PRDX data are limited. General biomarker literature outside COVID shows serum PRDX4 can reflect systemic OS and worse outcomes in several conditions, suggesting plausibility; a small pediatric study proposes serum PRDX4 could help distinguish acute COVID-19 from MIS-C, but this is preliminary and not yet a validated prognostic tool for adult severity/mortality (Schulte, 2011; El-Ghany et al., 2024).

Current COVID-19 genetics syntheses do not report PRDX gene variants among confirmed severity loci; transcriptomic studies do show peroxiredoxin-system activation (e.g., upregulation of SRXN1, which repairs hyperoxidized PRDXs), but germline PRDX SNP links to severity remain unproven (Saheb Sharif-Askari et al., 2021; Cappadona et al., 2023).

### 4.2 Serum oxidative enzymes and their genetic polymorphisms, as biomarkers of COVID-19 severity and mortality

The main enzymatic systems responsible for generating free radicals and ROS include reduced NOX, xanthine oxidase (XO), nitric oxide synthase (NOS), and myeloperoxidase (MPO). Their serum activity roles as prognostic biomarkers are summarized in Table 1, while their associated SNPs are presented in Table 2 and Figure 5 (Akira et al., 2006). Finally, Table 3 and Figure 6 present the gene structure, including exon and intron counts, SNP positions, and ClinVar annotations.

#### 4.2.1 NADPH oxidase enzyme

NOX enzymes catalyze the production of superoxide anion (O_2_^−•^). O_2_^−•^ is a powerful oxidizing agent and is considered the precursor of most ROS, which is highly reactive with water (H_2_O) molecules (Navarro-Yepes et al., 2014). These enzymes (NOX1–5, DUOX1/2), located in cellular and phagosomal membranes, convert superoxide into H_2_O_2_ and water, contributing to OS. NOX activation is proposed as a shared pathogenic mechanism underlying major comorbidities—such as hypertension, diabetes, and cardiovascular disease—that predispose individuals to severe COVID-19. Importantly, endosomal NOX activity is crucial for SARS-CoV cell entry, and elevated serum biomarkers of NOX activation have been observed in COVID-19 patients, reinforcing the enzyme’s role in disease progression (Violi et al., 2020). It is possible that the upregulation of this enzyme in COVID-19 is related to a rise in Ang concentrations and a reduction in ACE2. When SARS-CoV-2 binds to ACE2, it is unable to switch Ang Ⅱ to Ang one to seven, thus the amount of Ang Ⅱ will rise (Suhail et al., 2020). However, no peer-reviewed research to date explores whether genetic variants in these NOX components influence the clinical outcomes of SARS-CoV-2 infection.

#### 4.2.2 Xanthine oxidase enzyme

This enzyme metabolizes hypoxanthine, xanthine, and NADH to form O_2_^−•^ and H_2_O_2_. It has been involved in the pathogenesis of COVID-19 through two proposed mechanisms: First, it induces endothelial dysfunction through increasing O_2_^−•^ and decreases the bioavailability of NO. Second, it enhances neutrophil extracellular trap (NET)-related coagulopathy via stimulation of neutrophils to release NET (Pratomo et al., 2021). While serum XO activity has not been empirically validated as a biomarker for COVID-19 severity, its metabolic output—xanthine—has been identified through metabolomic profiling as a promising prognostic biomarker (Karvelsson et al., 2025). As part of antiviral response pathways, XO enzyme has been proposed as a therapeutic target (via inhibitors such as allopurinol or febuxostat) in the context of SARS-CoV-2 infection (Pratomo et al., 2021). There’s limited evidence directly linking *XO* gene polymorphisms to COVID-19 severity. Studies have explored the impact of inflammatory gene polymorphisms on COVID-19, but specific research on XO gene variations and their direct correlation with disease outcome is scarce.

#### 4.2.3 Myeloperoxidase enzyme

MPO is involved in the production of hypochlorous acid (HOCl), O_2_^−•^ and H_2_O_2_ (Figure 4). It has been related to vascular wall damage in pathophysiological situations such as COVID-19. Moreover, HOCl leads to degradation of hemoglobin and releases free iron in the form of ferrous ion (Fe^2+^) into the bloodstream, which in turn enters the Fenton reaction to enhance the production of •OH (Abu-Soud et al., 2014). While the serum level of MPO is elevated in non-hospitalized COVID-19 patients compared HCs, it does not experience significant change between hospitalized COVID-19 and HCs (Gelzo et al., 2021).

Some studies have revealed MPO polymorphisms impacting circulating MPO levels. A polymorphism at −129 G/A in the MPO gene was associated with decreased MPO serum concentration, and another at −463 G/A linked to lipid level changes (Hoy et al., 2001). There’s currently no published evidence suggesting that MPO gene variants influence COVID-19 severity or outcomes. While certain MPO polymorphisms have been tied to inflammatory or vascular conditions, their relevance to COVID-19 remains unexplored.

#### 4.2.4 Nitric oxide synthase enzyme

There are three isoforms of NOS enzyme, called neuronal NOS (nNOS), induced NOS (iNOS) and endothelial NOS (eNOS). Under physiological conditions, NO is synthesized from L-arginine, but under conditions of substrate or cofactor deficiency, there is a decrease in NO and an increase in O_2_^−•^ and peroxynitrite (ONOO-) (Landmesser et al., 2003). In addition, the eNOS has been related to the release of ROS from the mitochondrial electron transport chain into the cytoplasm in pathological situations (Bonomini et al., 2008). Endothelial nitric oxide synthase (eNOS) produces NO, a critical molecule for vascular health. Tetrahydrobiopterin (BH_4_) is a vital cofactor that ensures eNOS operates efficiently. When BH_4_ is deficient, eNOS becomes “uncoupled”—instead of producing NO, it generates superoxide (O_2_•^-^), a type of ROS (Kuzkaya et al., 2003).

NO is highly unstable and has a very short half-life in biological systems, making direct measurement impractical. Instead, its stable oxidation products—nitrate (NO_3_^−^) and nitrite (NO_2_^−^)—are quantified in blood, urine, or other samples as indirect indicators of NO production (Giustarini et al., 2008). Lorente et al. (2022) reported that critically ill COVID-19 patients with higher serum nitrate levels at ICU admission had significantly greater 30-day mortality, independent of APACHE-II and SOFA severity scores, suggesting nitrates as a potential prognostic biomarker (Lorente et al., 2022).

There is currently no direct evidence linking specific polymorphisms in the inducible nitric oxide synthase (iNOS or NOS2) and NOS1 genes to the severity of COVID-19. Although iNOS is a key enzyme in the inflammatory and antiviral response, with its expression elevated during SARS-CoV-2 infection, existing genetic association studies and reviews have not reported any NOS1 and NOS2 variants (e.g., SNPs) as being significantly correlated with COVID-19 severity or outcomes.

In contrast, most research on NOS polymorphisms in COVID-19 has focused on the endothelial isoform (eNOS/NOS3), with some studies suggesting that variants in NOS3 may influence disease course in specific populations—particularly younger individuals without comorbidities—via effects on vascular and OS pathways. The NOS3 rs2070744 (T–786C) promoter variant leads to reduced eNOS transcription and diminished NO production, impairing endothelial antioxidant and vasodilatory functions. This pro-oxidative imbalance is implicated in heightened cardiovascular risk: individuals with the CC genotype face increased mortality in heart failure and elevated susceptibility to conditions like coronary artery disease and myocardial infarction. A 2025 study examined COVID-19 patients and observed no significant association between the rs1799983 (G894T) genotypes or alleles and disease severity (p = 0.85 for genotypes; p = 0.78 for alleles). Meanwhile, another NOS3 polymorphism—the 27-bp VNTR (4b/a)—showed a trend toward association in younger patients (≤50 years) without COPD, with the 4b allele and 4b/4b genotype more frequent in severe cases (p values around 0.06–0.07), though these findings did not reach conventional statistical significance.

### 4.3 Master regulators of antioxidant response and their genetic polymorphism as biomarkers of COVID-19 severity

NFE2L2 (NRF2) encodes the master redox transcription factor NRF2, which binds AREs to induce cytoprotective genes (e.g., HMOX1/HO-1, GCL, NQO1); in COVID-19, multiple groups show suppression of NRF2 signalling in patient tissues and models, and that pharmacologic NRF2 activation (e.g., dimethyl fumarate, 4-octyl-itaconate) exerts antiviral and anti-inflammatory effects, linking weaker NRF2 tone to worse disease biology. Serum readouts downstream of NRF2—HO-1 protein, and related heme-handling biomarkers (heme, hemopexin), as well as bilirubin/ferritin—track with severity and outcomes and have been proposed as prognostic biomarkers in hospitalized patients. Host genetics also matter: in Mexican COVID-19 pneumonia, NFE2L2 rs2364723 (C>G) showed a protective association against severe disease, while variants in its repressor partner KEAP1 associated with more aggressive courses; more broadly, functional NRF2 promoter SNPs (rs6721961, rs6706649, rs35652124) modulate NRF2 expression and have been tied to disease risk in other oxidative-stress phenotypes, providing biologic plausibility for prognostic use in COVID-19 (Olagnier et al., 2020; Qu et al., 2023).

KEAP1 (Kelch-like ECH-associated protein 1) is the cytosolic sensor that targets NRF2 for ubiquitin-mediated degradation; oxidative or electrophilic stress disables KEAP1, allowing NRF2 to accumulate and transactivate defense genes. In SARS-CoV-2 infection, the virus can actively blunt the NRF2/HO-1 axis—for example, via NSP14, which impairs SIRT1-mediated NRF2 activation—potentially tilting patients toward hyper-inflammation and oxidative injury. A study of 110 COVID-19 pneumonia patients (51 severe, 59 moderate) and 111 controls found that OS regulation genes NFE2L2 (NRF2) and KEAP1 influence disease progression. Researchers genotyped NFE2L2 rs2364723C>G and KEAP1 rs9676881A>G and rs34197572C>T, using severity classification by ventilatory status and Berlin ARDS criteria. The G allele of NFE2L2 rs2364723 was significantly less frequent in severe cases, suggesting a protective effect against severe disease (p = 0.02), whereas the G allele of KEAP1 rs9676881 was more common in moderate cases (p = 0.04), and the T allele of KEAP1 rs34197572 was strongly associated with more aggressive COVID-19 both in severe vs. control (p = 0.001) and severe vs. moderate (p = 0.004) comparisons. These findings indicate that specific NRF2/KEAP1 polymorphisms may act as genetic prognostic biomarkers for COVID-19 severity through their role in OS regulation (Soto et al., 2022).

BACH1 (BTB and CNC homology 1) is a heme-responsive transcriptional repressor that antagonizes NRF2 on ARE/MARE enhancers, directly repressing HO-1; heme binding inactivates BACH1, relieving repression and permitting robust HO-1 induction. While BACH1-specific COVID-19 SNP data are lacking, the pathway is mechanistically relevant in SARS-CoV-2: HO-1 induction (a BACH1-controlled node) displays antiviral and cytoprotective activity, and multiple clinical and experimental datasets position HO-1 (and related heme-axis biomarkers) as severity/outcome biomarkers—supporting the idea that individuals with BACH1/NRF2 settings favoring higher HO-1 may fare better (Sun et al., 2002; Hou et al., 2008).

### 4.4 Biomarkers of oxidative damage

**FIGURE 7:**
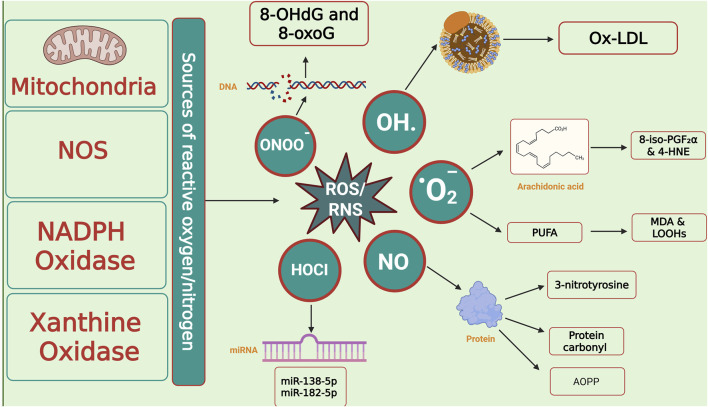
Cellular sources of reactive species and oxidative damage biomarkers. Mitochondria, nitric oxide synthase (NOS), NADPH oxidase, and xanthine oxidase represent the major enzymatic and cellular sources of reactive oxygen and nitrogen species (ROS/RNS), including superoxide (O_2_•^-^), hydroxyl radical (•OH), nitric oxide (NO•), hypochlorous acid (HOCl), and peroxynitrite (ONOO^−^). These reactive intermediates attack biomolecules, generating characteristic oxidative stress biomarkers: DNA lesions (8-OHdG, 8-oxoG), lipid peroxidation products (Ox-LDL, MDA, LOOHs, 8-iso-PGF_2_α, 4-HNE), and protein oxidation biomarkers (3-nitrotyrosine, protein carbonyls, AOPP). ROS/RNS also modulate epigenetic regulators such as microRNAs (e.g., miR-138-5p, miR-182-5p), linking oxidative stress to altered gene expression. Abbreviations: ROS: Reactive Oxygen Species; ONOO^−^: Peroxynitrite; OH·: Hydroxyl Radical; O_2_^−^: Superoxide Anion; HOCl: Hypochlorous Acid; NO: Nitric Oxide; NOS: Nitric Oxide Synthase; NADPH Oxidase: Nicotinamide Adenine Dinucleotide Phosphate Oxidase; 8-OHdG: 8-Hydroxy-2′-Deoxyguanosine; 8-oxoG: 8-Oxoguanine; Ox-LDL: Oxidized Low-Density Lipoprotein; PUFA: Polyunsaturated Fatty Acids; MDA: Malondialdehyde; LOOHs: Lipid Hydroperoxides; 8-iso-PGF2α: 8-Iso-Prostaglandin F2α; 4-HNE: 4-Hydroxy-2-Nonenal; AOPP: Advanced Oxidation Protein Products; miRNA: MicroRNA; 3-nitrotyrosine: nitrated tyrosine residue (biomarker of nitrosative stress). The image was created using the BioRender program, available at https://www.biorender.com.

OS biomarkers can be categorized into indicators of lipid, DNA, and protein damage. Their applications as prognostic biomarkers are summarized in Table 4 and illustrated in Figure 7.

**TABLE 4:** Oxidative stress damage biomarkers in COVID-19.

Marker	Definition	Measurement/ Method	COVID-19 associations	Prognostic value	Reference(s)	Sample size	Country
MDA	Reactive aldehyde byproduct of lipid peroxidation of PUFAs	TBARS assay or ELISA.	Elevated in COVID-19 patients vs. controls; higher in non-survivors	High levels linked with increased mortality	(Avila-Nava et al., 2022; Özenç et al., 2024)	152 (76 COVID-19 + 76 controls); 50 patients	Mexico; Turkey
4-HNE	Reactive α,β-unsaturated aldehyde; forms protein adducts	ELISA or MS.	Elevated in survivors (∼5.5 pmol/mg) and non-survivors (∼5.9 pmol/mg)	Persistent elevation in non-survivors = poor prognosis	Žarković et al. (2021)	21 patients (11M, 10F)	Croatia
8-iso-PGF_2_α	Stable prostaglandin-like compound from lipid peroxidation	Plasma/urine ELISA or MS.	Elevated in COVID-19 (esp. co-infection)	Linked to severity and death in CAP; likely same in COVID-19	(Muhammad et al., 2020; Di Minno et al., 2022; Zheng et al., 2021)	74 (20 controls +54 COVID-19); 52 (40 prostate cancer +12 controls); 96 (22 mild +74 severe)	Nigeria; Italy; China
LOOHs	Early products of lipid peroxidation; precursors to MDA/4-HNE.	FOX assay, spectrophotometric	Elevated in severe vs. mild COVID-19; correlates with inflammation	Predictive of ARDS and mortality	Yang et al. (2024)	N/A	China
oxLDL/oxLDL:LDL-C ratio	Oxidized LDL particles causing endothelial dysfunction	Immunoassays	Rises stepwise from non-severe → critical; correlates with CRP.	Independent risk factor; day 0→6 rise predicts death	(Pelin et al., 2023; Liu et al., 2024)	187 patients; 200 (100 COVID-19 + 100 controls)	China; Turkey
8-OHdG	Modified nucleoside from oxidative DNA damage	ELISA or chromatography	Elevated in COVID-19; higher in severe vs. moderate cases	Predicts outcome; correlates with severity	(Satała et al., 2023; Domínguez-de-Barros et al., 2025)	72 patients; 105 (75 COVID-19 + 30 controls)	Poland; Spain
8-oxoG	Oxidized guanine base in DNA.	Leukocyte assays	Elevated in leukocytes; repair enzymes upregulated	Indicates DNA injury and repair response	Olsen et al. (2022)	Cohort 1: 22 (8-oxoG), 17 (BER), 10 controls; Cohort 1 GE: 15 + 5 controls; Cohort 2 GE: 15 + 6 controls	Norway
Protein Carbonyls	Oxidized proteins forming carbonyl groups	DNPH assay at 370 nm	Increased in COVID-19 plasma vs. controls; higher in severe and pregnant	Reflects severity and disease progression	(Žarković et al., 2022); Aryal et al. (2023)	88 patients; 60 (10 acute, 30 convalescent, 20 controls)	Croatia; United States of America
3-NT	Nitration of tyrosine residues by RNS (e.g., peroxynitrite)	ELISA or LC-MS/MS.	Elevated in moderate to severe COVID-19; correlates with inflammation	Supports progression assessment	Liu et al. (2024)	187 patients	China
AOPP	Cross-linked protein fragments via chlorinated oxidants	ELISA.	Higher in severe vs. moderate COVID-19; correlates with neutrophils, vWF, D-dimer	Elevated in survivors vs. non-survivors; prognostic	Satała et al. (2023)	72 patients	Poland
miR-182-5p and miR-138-5p	Circulating microRNAs linked to oxidative stress	RT-qPCR.	In mild COVID-19: miR-182-5p correlates with MDA; miR-138-5p with 8-OHdG	Early biomarker; promising prognostic tool	Domínguez-de-Barros et al. (2025)	105 (75 COVID-19 + 30 controls)	Spain

Biomarkers listed represent key oxidative stress–related damage indicators measured in COVID-19, patients. Abbreviations: MDA, malondialdehyde; TBARS, thiobarbituric acid reactive substances; ELISA, enzyme-linked immunosorbent assay; FiO_2_, fraction of inspired oxygen; 4-HNE, 4-hydroxynonenal; iso-PGF_2_α, 8-iso-prostaglandin F_2_α; CAP, community-acquired pneumonia; LOOHs, lipid hydroperoxides; FOX, ferrous oxidation–xylenol orange; oxLDL, oxidized low-density lipoprotein; LDL-C, low-density lipoprotein cholesterol; CRP, C-reactive protein; 8-OHdG, 8-hydroxy-2′-deoxyguanosine; 8-oxoG, 8-oxo-guanine; OGG1, 8-oxoguanine DNA, glycosylase 1; APE1, apurinic/apyrimidinic endonuclease 1; DNPH, 2,4-dinitrophenylhydrazine; 3-NT, 3-nitrotyrosine; LC-MS/MS, liquid chromatography tandem mass spectrometry; AOPP, advanced oxidation protein products; HOCl, hypochlorous acid; vWF, von Willebrand factor; DNP, dinitrophenyl; Alb, albumin; miR, microRNA; RT-qPCR, reverse transcription quantitative polymerase chain reaction; ARDS, acute respiratory distress syndrome.

#### 4.4.1 Lipid peroxidation products

MDA (often measured by TBARS), 4-HNE, 8-iso-PGF_2_α, lipid hydroperoxides (LOOHs), and oxidized LDL (oxLDL)—arise when polyunsaturated lipids are attacked by ROS; 4-Hydroxynonenal (4-HNE) and MDA form adducts with proteins/DNA, while isoprostanes (e.g., 8 iso PGF2α) are prostaglandin like end products considered highly reliable *in vivo* biomarkers. In COVID-19, several cohorts show higher MDA and NO metabolites in severe disease, oxLDL and the oxLDL/LDL C ratio rising stepwise from non-severe, severe, critical illness, and elevated 8-iso-PGF_2_α in infected patients (and in some settings co infection), all supporting a link between lipid oxidation and worse clinical courses. Importantly, composite panels of oxidative biomarkers can predict mortality in hospitalized patients.

##### 4.4.1.1 Malondialdehyde as lipid peroxidation biomarkers

MDA is a byproduct of the decomposition of unsaturated lipids which occurs when ROS attack polyunsaturated fatty acids, primarily derived from arachidonic acid in the cellular membrane, leading to the formation of MDA. Tissue damage can increase MDA levels, and MDA can react with lysine residues, producing protein alterations that trigger mechanisms. A study conducted in Mexico has shown a strong link between elevation of MDA and COVID-19 mortality (Avila-Nava et al., 2022).

##### 4.4.1.2 4-Hydroxynonenal

4-HNE is a highly reactive α,β-unsaturated aldehyde generated during lipid peroxidation of polyunsaturated fatty acids. It forms stable adducts with proteins (e.g., histidine residues), serving as a sensitive indicator of oxidative damage. In patients with COVID-19, elevated plasma 4-HNE–protein adduct levels have been observed in both survivors and non-survivors—averaging around 5.5 pmol/mg and 5.9 pmol/mg protein *versus* ∼3 pmol/mg in healthy controls—highlighting systemic OS in infection. Furthermore, dynamic measurements revealed that survivors demonstrated fluctuating adduct levels, while non-survivors showed persistently high 4-HNE levels, correlating with fatal outcomes. Studies also link increased lipid peroxidation biomarkers such as 4-HNE and MDA with a significantly higher risk of 28-day intubation or death in COVID-19 patients, underlining their prognostic relevance in severe disease (Žarković et al., 2024).

##### 4.4.1.3 8-Isoprostane

8-iso-PGF_2_α, a chemically stable prostaglandin-like compound formed via non-enzymatic free radical peroxidation of arachidonic acid, is considered one of the most reliable *in vivo* biomarkers of lipid peroxidation. Recent research indicates that circulating 8-iso-PGF_2_α levels are significantly elevated in patients with severe COVID-19 compared to those with milder disease and healthy controls. For example, a cross-sectional study from Türkiye reported notably higher serum 8-iso-PGF_2_α in patients with severe COVID-19 than in mild or control subjects, with significant positive correlations between 8-iso-PGF_2_α and disease progression biomarkers, suggesting that elevated lipid peroxidation is linked to clinical severity. These findings support the utility of 8-iso-PGF_2_α not only as an OS indicator but also as a potential prognostic biomarker in COVID-19 (Kurutas et al., 2024).

##### 4.4.1.4 Isofurans, neuroprostanes, and neurofurans

Isofurans, neuroprostanes, and neurofurans are advanced lipid peroxidation biomarkers that reflect OS in specific biological contexts—among them, neurological injury. Isofurans arise nonenzymatically from arachidonic acid, especially under elevated oxygen conditions, while neuroprostanes and neurofurans derive from docosahexaenoic acid and adrenic acid within nervous tissue, offering CNS-specific insights into oxidative damage (Humaloja et al., 2021). Despite the well-documented role of OS in COVID-19 pathogenesis, including in neurological complications, these molecules have not yet been studied or measured in the context of SARS-CoV-2 infection, leaving a notable gap in the understanding of CNS lipid peroxidation in COVID-19.

##### 4.4.1.5 Lipid hydroperoxides

LOOHs are the primary molecular products generated early during lipid peroxidation when ROS abstract hydrogen atoms from polyunsaturated fatty acids in cell membranes, forming peroxyl radicals that quickly yield LOOHs. Though inherently unstable, LOOHs propagate oxidative chain reactions and decompose into secondary reactive aldehydes like malondialdehyde and 4-HNE. In COVID-19, LOOHs are markedly elevated in patients with severe pneumonia compared to mild cases or healthy controls, reflecting widespread oxidative membrane damage. Notably, higher LOOH levels correlate with increased inflammatory biomarkers and are predictive of poorer clinical outcomes, including progression to acute respiratory distress syndrome and mortality. These findings suggest LOOHs are both a mechanistic and prognostic indicator of oxidative injury in SARS-CoV-2 infection (Žarković et al., 2021).

##### 4.4.1.6 Oxidized low-density lipoprotein

oxLDL is generated when LDL’s lipid core and apoB are oxidatively modified, yielding pro-inflammatory particles that impair endothelial function; in COVID-19, serum oxLDL and the oxLDL/LDL-C ratio rise stepwise from non-severe to severe/critical illness and correlate with inflammation (e.g., CRP), and oxLDL acts as an independent risk factor for progression to severe disease. Moreover, in a prospective cohort, oxLDL showed the strongest discrimination between COVID-19 patients and healthy controls among oxidative-stress biomarkers, and longitudinal profiling in hospitalized patients found that an increase in oxLDL from day 0 to day 6 above the 90th percentile predicted in-hospital death (all patients above this threshold died), linking oxLDL dynamics to mortality risk. Taken together, oxLDL is both a mechanistic biomarker of oxidative lipid injury and a clinically useful biomarker for severity stratification and outcome prediction in SARS-CoV-2 infection (Žarković et al., 2022).

#### 4.4.2 DNA damage biomarkers

DNA damage biomarkers chiefly 8-hydroxy-2′-deoxyguanosine (8-OHdG, also called 8-oxo-dG) and 8-Oxoguanine (8-oxoG) reflect oxidative modification of guanine in nuclear or mitochondrial DNA (with related lesions such as isoguanine, 8 oxoadenine, 5 hydroxycytosine, and halogenated bases also reported). In COVID-19, higher circulating oxidative nucleic acid damage associates with organ dysfunction and in hospital mortality, and independent cohorts report higher 8-OHdG in severe vs. moderate cases.

##### 4.4.2.1 8- hydroxy-2′-deoxyguanosine

8-OHdG is a biomarker of oxidative DNA damage, reflecting the impact of ROS on DNA molecules, which can result in mutations and genomic instability (Urbaniak et al., 2020). It was documented that the level of urinary 8-OHdG was significantly higher among SARS-CoV2-infected patients compared to healthy control and its level positively correlated with the severity of infection and it could be a potential biomarker for OS in COVID-19 patients (Saeed and Najeeb, 2023).

In a longitudinal cohort study of COVID-19 patients, plasma 8-OHdG was measured alongside other oxidative damage biomarkers such as MDA and advanced oxidation protein products (AOPP). The findings revealed a significant rise in 8-OHdG levels on day 7 after admission relative to baseline (p < 0.005), even though levels initially did not differ significantly from controls at admission. This increase coincided with elevated inflammatory parameters and biomarkers of organ dysfunction, such as ALT and creatinine, suggesting that oxidative DNA damage correlates with systemic injury (Coric et al., 2021a). However, in another cross-sectional cohort spanning non-severe, severe, and critically ill COVID-19 patients, 8-OHdG levels did not differ significantly across severity categories—highlighting variability possibly due to study design or disease phase (Liu et al., 2024).

##### 4.4.2.2 8-oxoguanine

8-oxoG is a mutagenic oxidized guanine base generated when ROS attack DNA, while 8-hydroxy-2′-deoxyguanosine is its deoxynucleoside form, released into circulation and excreted in urine following base excision repair by enzymes such as 8-oxoguanine DNA glycosylase (OGG1). Both are widely recognized biomarkers of oxidative DNA damage, with 8-oxoG reflecting intracellular DNA injury and 8-OHdG serving as a stable systemic biomarker. A study by Olsen et al. (2022) examined oxidative DNA damage, including 8-oxoG, in blood cells of patients infected with SARS-CoV-2. The authors reported significantly elevated levels of 8-oxoG in leukocytes from hospitalized COVID-19 patients compared to healthy controls, suggesting heightened intracellular oxidative DNA injury. Importantly, the study also found upregulation of repair enzymes such as OGG1 and APE1, indicating activation of the base-excision repair pathway in response to this damage. These findings underscore the heightened oxidative burden in COVID-19 and the cellular response to repair 8-oxoG lesions.

#### 4.4.3 Protein oxidation/nitration product

Protein oxidation/nitration readouts include protein carbonyls and 3-nitrotyrosine (3-NT) (a nitrative stress biomarker driven by ONOO-) and AOPP which frequently track with severity, complementing lipid/DNA biomarkers.

##### 4.4.3.1 Protein carbonyl

Protein carbonylation is a biomarker of protein oxidation, where proteins are modified by ROS, leading to altered protein structure and function. Aryal et al. (2023) reported that COVID-19 patients exhibit elevated levels of protein oxidative damage, specifically carbonylated proteins, indicating increased OS levels during the disease. Furthermore, other research highlighted the positive correlation between protein oxidative damage and disease severity in COVID-19 (Spencer et al., 2022).

##### 4.4.3.2 3-nitrotyrosine

3-NT, a product of tyrosine nitration by RNS like ONOO-, is a well-established biomarker of nitrosative stress and has been shown to correlate with COVID-19 severity. Elevated levels of 3-NT have been observed in patients with moderate to severe COVID-19 symptoms compared to those with milder disease, reflecting increased oxidative and nitrosative stress associated with systemic inflammation and endothelial dysfunction. A 2024 study in Scientific Reports reported significantly higher 3-NT levels in severe cases, suggesting its potential as a biomarker for disease progression and severity assessment (Wolszczak-Biedrzycka et al., 2024b). This aligns with broader findings that OS contributes to COVID-19 pathophysiology, supporting the role of 3-NT in monitoring inflammatory and redox-related complications (Dominic et al., 2021).

##### 4.4.3.3 Advanced oxidation protein products

AOPPs are dityrosine-containing, cross-linked protein fragments generated when plasma proteins, such as albumin, fibrinogen, and lipoproteins are modified by chlorinated oxidants (like HOCl) via MPO activity, making them reliable biomarkers of oxidative protein damage and systemic OS (Perrone et al., 2019). In the context of COVID-19, elevated AOPP levels have been associated with increased disease severity (Satała et al., 2023). A 2023 study found significantly higher serum AOPP concentrations in individuals with severe COVID-19 compared to those with moderate disease, and these levels correlated positively with biomarkers like neutrophil counts, von Willebrand factor, D-dimer, and glucose in severe cases (Satała et al., 2023). Similarly, a French cohort study evaluating oxidative biomarkers in hospitalized COVID-19 patients reported that while AOPP levels rose significantly in certain severity stages (notably stages 1 and 3), other biomarkers like thiols decreased, and ischemia-modified albumin (IMA) increased, suggesting a complex OS profile in more severe disease (Ducastel et al., 2021). Moreover, AOPP levels have demonstrated prognostic value: in a Brazilian cohort of severely symptomatic COVID-19 patients, AOPP levels differed significantly between survivors and non-survivors, alongside other oxidative biomarkers such as α-tocopherol and GSH (Neves et al., 2023).

#### 4.4.4 miRNAs as novel biomarkers of oxidative stress

Recent research highlights specific circulating microRNAs (miRNAs) as promising early biomarkers of OS in mild COVID-19 cases (Table 5). In a study analyzing non-invasive samples, miR-182-5p was found to correlate positively with MDA levels (r = 0.582, p = 0.01), reflecting lipid peroxidation activity, while miR-138-5p expression showed a significant association with oxidative DNA damage biomarker 8-OHdG (r = 0.403, p = 0.05). Notably, miR-138-5p was significantly upregulated in COVID-19 cases compared to healthy controls, indicating a potential antiviral and damage-response role. Conversely, miR-210-3p—a hypoxia- and OS–responsive miRNA—was downregulated in infected individuals. These findings suggest that miR-182-5p and miR-138-5p reflect early oxidative cellular injury and could serve as non-invasive, accessible biomarkers to monitor disease impact and guide prognosis even in mild SARS-CoV-2 infection (Domínguez-de-Barros et al., 2025). In the study by Domínguez-de-Barros et al., while miR-34a-5p and miR-155-5p expression was not altered in mild COVID-19 cases, miR-155 is frequently discussed in the COVID-19 literature as an oxidative/inflammatory miRNA biomarker (Domínguez-de-Barros et al., 2025).

**TABLE 5:** Circulating microRNAs Associated with Oxidative Stress Markers in Mild COVID-19 Patients.

microRNA	Expression in COVID-19 vs. Controls	Correlated OS marker	Correlation (R, p-value)	Biological/Pathophysiological insight	References
miR-182-5p	NS	Malondialdehyde (MDA, lipid peroxidation marker)	r = 0.582 p = 0.01	Reflects lipid peroxidation activity; indicates oxidative cellular injury	Domínguez-de-Barros et al. (2025)
miR-138-5p	↑	8-hydroxy-2′-deoxyguanosine (8-OHdG, oxidative DNA damage marker)	r = 0.403 p = 0.05	Suggests antiviral and damage-response role; marker of oxidative DNA injury	Domínguez-de-Barros et al. (2025)
miR-210-3p	↓	Hypoxia- and OS–responsive marker	NS	Indicates impaired hypoxia/oxidative stress response; altered redox adaptation	Domínguez-de-Barros et al. (2025)
miR-21-5p	↑ (higher in critical/fatal cases)	OS-inflammation biology (NOX/NRF2 axis, not a single marker)	Not evaluated	Prognostic signal for severity/mortality; tightly linked to redox-inflammatory pathways	Liang et al. (2023)
miR-146a-5p	↓ (notably in severe disease; also linked to poor response to anti-IL-6 therapy)	OS-inflammation regulation (NF-κB/NRF2 crosstalk)	Not evaluated	Low levels associate with worse outcomes and dysregulated redox-inflammation	Keikha et al. (2023)
miR-155-5p	↑	Reported in literature as associated with OS	NS	Inflammatory miRNA often upregulated in oxidative/inflammatory states; potential biomarker of redox-inflammatory interplay	Domínguez-de-Barros et al. (2025)

OS, oxidative stress; miRNA, microRNA; MDA, malondialdehyde; 8-OHdG, 8-hydroxy-2′-deoxyguanosine; R, correlation coefficient; p, probability value; NOX, NADPH, oxidase; NRF2, nuclear factor erythroid 2–related factor 2; NF-κB, nuclear factor kappa-light-chain-enhancer of activated B cells; IL-6, interleukin-6.

↑, upregulated; ↓, downregulated; NS, not significant.

Emerging evidence highlights the prognostic potential of miR-21-5p and miR-146a-5p in COVID-19, particularly in the context of oxidative stress and inflammation. miR-21-5p is consistently upregulated, with markedly higher expression in critical and fatal cases, and is closely associated with redox-inflammatory pathways involving the NOX/NRF2 axis. This suggests its utility as a biomarker of poor prognosis and mortality risk (Liang et al., 2023). Conversely, miR-146a-5p is significantly downregulated in severe COVID-19 and has also been linked to poor response to anti-IL-6 therapy. Functionally, reduced miR-146a-5p impairs regulation of NF-κB/NRF2 crosstalk, contributing to persistent inflammation and redox imbalance (Keikha et al., 2023). Together, these findings indicate that miR-21-5p (↑) and miR-146a-5p (↓) may serve as complementary biomarkers, reflecting the dysregulated oxidative and inflammatory responses that underlie disease progression and treatment resistance in severe COVID-19.

### 4.5 Antioxidant cofactors as prognostic biomarkers

**FIGURE 8:**
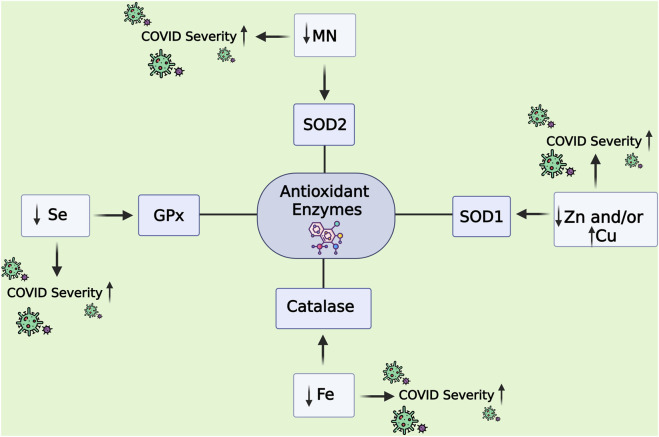
Antioxidant enzyme cofactors as prognostic biomarkers in COVID-19 severity. The figure illustrates how trace element cofactors regulate the activity of key antioxidant enzymes and how their deficiency or imbalance contributes to COVID-19 progression. Manganese (Mn) supports SOD2 activity, while zinc (Zn) and copper (Cu) are essential for SOD1. Selenium (Se) is a cofactor for glutathione peroxidase (GPx), and iron (Fe) is required for catalase. Decreased levels of Se, Mn, and Fe, or dysregulated Zn and Cu balance, impair antioxidant defense mechanisms, thereby increasing oxidative stress and contributing to enhanced COVID-19 severity. Abbreviations: GPx: Glutathione Peroxidase; SOD1: Superoxide Dismutase 1; SOD2: Superoxide Dismutase 2; Catalase: Hydrogen Peroxide-Catalyzing Enzyme; Se: Selenium; Mn: Manganese; Zn: Zinc; Cu: Copper; Fe: Iron. The image was created using the BioRender program, available at https://www.biorender.com.

Cofactors such as Zn, Cu, Se, and Fe, used as prognostic biomarkers, are presented in Table 6 and illustrated in Figure 8. Se is an essential cofactor for the activity of GPx. Furthermore, SOD depends on Cu and Zn; the deficiency of Cu and Zn decreases the activity of SOD and increases the activity of cytochrome P-450, stimulating the production of ROS (Wallenberg et al., 2010). Severe deficiency of Se and Zn has been found in critical COVID-19 patients in six European countries (Demircan et al., 2022). Zn deficiency is associated with COVID-19 mortality, increasing hospital stay and oxygen supplementation (Skalny et al., 2021). Research has shown that, although Cu has antiviral properties, the levels of Cu are higher in COVID-19 patients and are linked to how severe the disease is (Skalny et al., 2021).

**TABLE 6:** Antioxidant enzyme cofactors as prognostic biomarkers.

Biomarker/Cofactor	Biological role/Definition	Measurement	COVID-19 associations	Prognostic value	Sample size	Country
Cu	Cofactor for Cu–Zn SOD1, ceruloplasmin, andcytochrome c oxidase	Serum/plasma Cu; trace-element panels	Copper levels needed to rise by 50% to show an association with disease severity, and this link emerged only after accounting for C-reactive protein levels—suggesting that copper may contribute to COVID-19 severity through its pro-inflammatory and pro-oxidant effects (Al-Saleh et al., 2022)	High Cu ratio associated with severity and systemic inflammation	48 countries	Diverse global sample including countries from Europe, Asia, Africa, and the Americas
Zn	Cofactor for Cu–Zn SOD and metallothioneins	Serum/plasma Zn; nutritional trace assay	The study clearly demonstrates that many COVID-19 patients had zinc deficiency, which was linked to a higher risk of complications, longer hospitalization, and greater mortality (Jothimani et al., 2020)	Low Zn levels predictive of worse outcomes	COVID-19 patients (n = 47) compared to healthy controls (n = 45)	India
Se	Cofactor for GPx and thioredoxin reductases	Serum/plasma Se; GPx activity assays	Researchers analyzed 166 serum samples from 33 COVID-19 patients, measuring total Se and the Se -carrying protein SELENOP. Compared to a large European reference population, COVID-19 patients showed significantly lower levels of both Se (50.8 vs. 84.4 μg/L) and SELENOP (3.0 vs. 4.3 mg/L). Alarmingly, over 40% of the samples were below the 2.5th percentile of the normal range (Moghaddam et al., 2020)	Survivors had significantly higher Se and SELENOP levels than non-survivors, with Se status improving over time only in those who recovered	33 COVID-19 patients (166 serum samples), compared against 1,915 subjects from the European EPIC reference cohort	Germany
Fe	Cofactor for CAT and MPO	Serum ferritin, Fe, TIBC; catalase activity	low serum Fe and transferrin levels are associated with poor outcomes, with specific thresholds (e.g., ferritin >635 ng/mL, serum Fe < 6 μmol/L) offering predictive accuracy for severe disease and death (Suriawinata and Mehta, 2023)	Decreased serum Fe, have strong prognostic value in COVID-19		
Manganese (Mn)	Cofactor for SOD2; critical in mitochondrial ROS clearance	Blood Mn; SOD2 enzyme activity (research use)	COVID-19 infection is associated with widespread trace element imbalance, notably deficiencies in Mn (Al-Fartusie et al., 2023)	Potential target via Mn-based SOD mimetics; prognostic utility not yet validated	120 Individuals: 40 COVID-19 patients; 40 recovered patients; 40 healthy controls	Iraq
Cu/Zn Ratio	Composite biomarker of redox and inflammatory balance (high Cu:Zn = high inflammation)	Calculated from serum Cu and Zn	Cu/Zn ratio may amplify inflammatory responses in COVID-19 patients and could work in tandem with other factors to worsen disease severity (Al-Saleh et al., 2022)	Predictive of severe disease, cytokine storm, and outcome trajectory	155 COVID-19 patients, grouped by severity 16 Asymptomatic 49 Mild 68 Moderate 22 Severe	Saudi Arabia

Antioxidant enzyme cofactors represent essential trace elements influencing redox homeostasis and immune response. Abbreviations: Cu, copper; Zn, zinc; Se, selenium; Fe, iron; Mn, manganese; SOD1/2, superoxide dismutase-1/2; CAT, catalase; MPO, myeloperoxidase; GPx, glutathione peroxidase; SELENOP, selenoprotein P; TIBC, total iron-binding capacity; ROS, reactive oxygen species; CRP, C-reactive protein. Elevated Cu or Cu/Zn ratios are linked to inflammation and poor prognosis, while deficiencies in Zn, Se, and Mn are associated with disease severity and unfavorable outcomes.

Iron plays a dual and complex role in the pathogenesis and prognosis of COVID-19. Biologically, iron is a critical cofactor for antioxidant and immune defense enzymes such as catalase, which decomposes H2O2, and MPO, which participates in neutrophil-mediated microbial killing—functions essential for managing OS and infection. However, during SARS-CoV-2 infection, iron homeostasis becomes severely disrupted. Pro-inflammatory cytokines upregulate hepcidin, reducing serum iron availability by sequestering it within macrophages and hepatocytes. This leads to hypoferremia, impairing immune function and oxygen transport, while ferritin, an iron storage protein and acute-phase reactant, becomes elevated—often dramatically. High ferritin levels have been consistently associated with worse clinical outcomes, including ICU admission and mortality, marking it as a strong prognostic indicator (Edeas et al., 2020; Vargas-Vargas and Cortés-Rojo, 2020). Likewise, low serum iron levels—particularly below 6 µmol/L—have been shown to predict hospitalization and adverse outcomes in COVID-19 patients (Suriawinata and Mehta, 2023). This paradoxical pattern of hyperferritinemia and hypoferremia reflects a maladaptive inflammatory response that amplifies OS and tissue damage, positioning iron not only as a key cofactor in antioxidant defense but also as a dynamic biomarker of COVID-19 severity.

Mn serves as an essential cofactor for mitochondrial superoxide dismutase (SOD2 or MnSOD), a critical enzyme that converts damaging mitochondrial superoxide radicals (O_2_•–) into H_2_O_2_ and oxygen, thereby protecting cellular integrity from OS. In the 2023 study by Al-Fartusie et al. (2023) found that Mn was significantly lower in the serum of COVID-19 patients compared to both recovered individuals and HCs (p < 0.0001). This deficiency suggests a disruption in antioxidant defences, as Mn is a critical cofactor for mitochondrial SOD2, an enzyme that protects cells from oxidative damage by converting superoxide radicals into less harmful molecules. The reduction in Mn may impair SOD2 activity, potentially contributing to the OS and inflammation seen in COVID-19. While Mn levels were restored in recovered individuals, the lack of significant difference between recovered and healthy groups further implies Mn depletion is specific to the active disease phase. Additionally, ROC curve analysis showed Mn had a good diagnostic performance (AUC = 0.872), with 92% sensitivity and 70% specificity at a cutoff value of 0.0145 μg/dL, highlighting its potential as a biomarker for COVID-19 susceptibility or active infection. However, the study notes that more research is needed to fully understand Mn’s role in immune response and COVID-19 severity.

### 4.6 Gene–gene interaction network of oxidative stress–related genes

#### 4.6.1 Gene–gene interaction network via GeneMANIA

**FIGURE 9:**
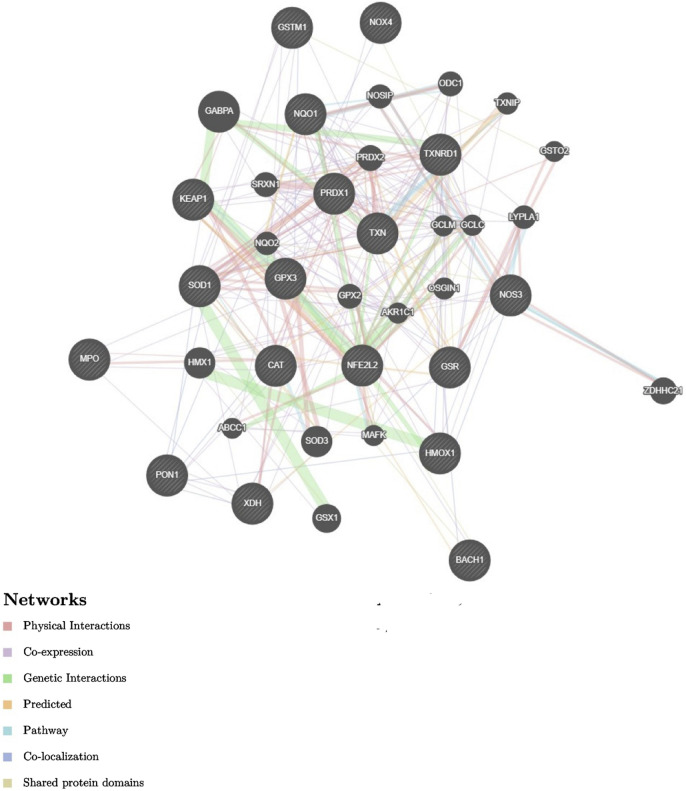
Gene–gene interaction network of oxidative stress–related genes (GeneMANIA). The network shows interactions among antioxidant enzymes (GPX3, GSR, GSTM1, SOD1, CAT, PRDX1, NQO1, TXN, TXNRD1, PON1, HMOX1), transcriptional regulators (NFE2L2/NRF2, KEAP1, BACH1), and pro-oxidant enzymes (NOX4, MPO, XDH, NOS3). Node colors: black = query genes, gray = predicted related genes. Edge colors: pink = co-expression, blue = physical interactions, green = genetic interactions, purple = pathway links, yellow = co-localization, orange = predicted interactions, teal = shared protein domains. The map highlights the balance between oxidant-producing and antioxidant systems in redox regulation. Abbreviations: CAT: Catalase; GPX1/2/3: Glutathione Peroxidase 1/2/3; GSTM1: Glutathione S-Transferase Mu 1; GSTO2: Glutathione S-Transferase Omega 2; GSR: Glutathione-Disulfide Reductase; GCLM/GCLC: Glutamate-Cysteine Ligase Modifier and Catalytic Subunits; HMOX1: Heme Oxygenase 1; KEAP1: Kelch-Like ECH-Associated Protein 1; MPO: Myeloperoxidase; NFE2L2: Nuclear Factor Erythroid 2-Like 2 (Nrf2); NOX4: NADPH Oxidase 4; NOS3: Nitric Oxide Synthase 3; NOSIP: Nitric Oxide Synthase Interacting Protein; ODC1: Ornithine Decarboxylase 1; PON1: Paraoxonase 1; PRDX1/2: Peroxiredoxin 1/2; SRXN1: Sulfiredoxin 1; SOD1/2/3: Superoxide Dismutase 1/2/3; TXN: Thioredoxin; TXNIP: Thioredoxin-Interacting Protein; TXNRD1: Thioredoxin Reductase 1; ABCC1: ATP-Binding Cassette Subfamily C Member 1; AKR1C1: Aldo-Keto Reductase Family 1 Member C1; GABPA: GA-Binding Protein Subunit Alpha; GSX1: GS Homeobox 1; LYPLA1: Lysophospholipase 1; MAFK: Small Maf Protein K; BACH1: BTB Domain and CNC Homolog 1; XDH: Xanthine Dehydrogenase; ZDHHC24: Zinc Finger DHHC-Type Palmitoyltransferase 24.

The gene–gene interaction network highlights the intricate interplay between classical antioxidant enzymes (such as GPX, CAT, SOD, PRDX, and GSR) and regulatory proteins including NFE2L2, KEAP1, and BACH1, which are central to the oxidative stress response. The visualization shows strong co-expression and pathway connectivity among glutathione-dependent enzymes (GPX3, GSTM1, GSR, NQO1) and thioredoxin-related proteins (TXN, TXNRD1), suggesting that these parallel systems work synergistically to maintain redox balance (Figure 9). This aligns with previous studies showing that glutathione- and thioredoxin-based systems act as complementary antioxidant networks, providing cellular defense against hydrogen peroxide and lipid peroxides.

Beyond enzymatic antioxidants, the network emphasizes the regulatory dominance of NFE2L2 (NRF2), a transcription factor controlling the antioxidant response element (ARE) pathway. Its connections with KEAP1 and BACH1 reflect a tightly regulated system in which NRF2 activation induces genes such as NQO1, HMOX1, and GCLC, while KEAP1 and BACH1 repress NRF2 activity under basal conditions. The clustering of these transcriptional regulators with enzymatic antioxidants suggests a central hub for redox-sensitive gene regulation, which has been implicated in disease states ranging from cancer and cardiovascular disease to viral infections such as COVID-19.

Interestingly, the network also integrates pro-oxidant enzymes including NOX4, MPO, XDH, and NOS3, which generate reactive oxygen or nitrogen species, positioning them in proximity to antioxidant defenses. This balance between oxidant production and detoxification is crucial for understanding redox homeostasis under pathological stress. The identified links to secondary regulators (e.g., TXNIP, SRXN1, GCLM, and OSGIN1) further support the hypothesis that adaptive responses to oxidative stress involve both canonical detoxifying enzymes and stress-induced signaling proteins. Overall, the network illustrates the cooperative yet competitive interactions between oxidant-generating and antioxidant systems, underscoring their relevance in oxidative stress–driven conditions such as inflammation, cardiovascular dysfunction, and viral pathogenesis.

#### 4.6.2 Gene–gene interaction network via Cytoscape Software

**FIGURE 10:**
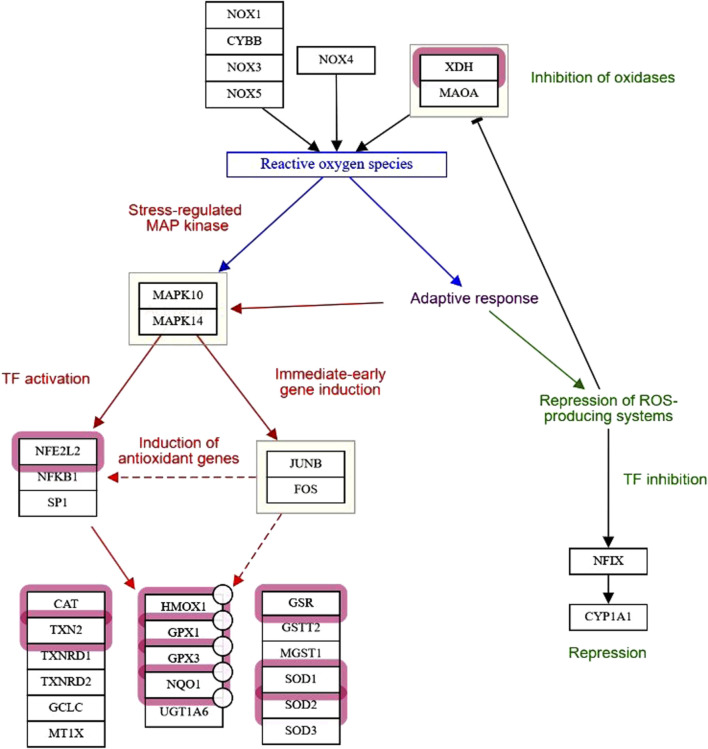
The oxidative stress response pathway showing gene interactions in ROS regulation and antioxidant defense. Blue arrows indicate ROS-driven adaptive signaling, red arrows represent MAP kinase–mediated gene induction, and green arrows denote inhibitory control of ROS-producing systems. Pink-highlighted genes (GPX1, GSR, CAT, SOD1/2, HMOX1, NFE2L2, and XDH) are study-specific query genes analyzed in Cytoscape, Version 3.10.3. Abbreviations: NOX1/3/4/5: NADPH Oxidase Isoforms 1/3/4/5; CYBB: Cytochrome b-245 Beta Chain; XDH: Xanthine Dehydrogenase; MAOA: Monoamine Oxidase A; ROS: Reactive Oxygen Species; MAPK10: Mitogen-Activated Protein Kinase 10 (JNK3); MAPK14: Mitogen-Activated Protein Kinase 14 (p38α); NFE2L2: Nuclear Factor Erythroid 2-Like 2 (Nrf2); NFKB1: Nuclear Factor Kappa B Subunit 1; SP1: Specificity Protein 1; JUNB: JunB Proto-Oncogene; FOS: Fos Proto-Oncogene; CAT: Catalase; TXN2: Thioredoxin 2; TXNRD1/2: Thioredoxin Reductase 1/2; GCLC: Glutamate-Cysteine Ligase Catalytic Subunit; MT1X: Metallothionein 1X; HMOX1: Heme Oxygenase 1; GPX1/3: Glutathione Peroxidase 1/3; NQO1: NAD(P)H Quinone Dehydrogenase 1; UGT1A6: UDP-Glucuronosyltransferase 1A6; GSR: Glutathione-Disulfide Reductase; GSTT2: Glutathione S-Transferase Theta 2; MGST1: Microsomal Glutathione S-Transferase 1; SOD1/2/3: Superoxide Dismutases 1/2/3; NFIX: Nuclear Factor I/X; CYP1A1: Cytochrome P450 Family 1 Subfamily A Member 1.

The gene interaction network was further visualized using Cytoscape Software (Version 3.10.3), highlighting the oxidative stress pathway and its regulatory complexity (Figure 10). This pathway integrates genes responsible for both ROS generation and antioxidant defense, ensuring redox homeostasis within cells. Core antioxidant enzymes, including GPX1–3, GSR, GST family members (e.g., GSTA1, GSTM1, GSTK1), SOD1/2, CAT, HMOX1/2, NQO1, TXN/TXN2, and PRDXs, play vital roles in detoxifying ROS and repairing oxidative damage. Their expression is primarily controlled by transcription factors such as NFE2L2 (NRF2), with KEAP1 and BACH1 acting as negative regulators. At the same time, ROS-producing genes such as DUOX1/2, NOXA1, XDH, NOS1–3, and MPO contribute to regulated ROS levels required for cellular signaling. The pathway is further influenced by stress-regulated kinases and immediate-early genes, enabling adaptive responses under oxidative conditions. Collectively, this interconnected gene network underscores the dynamic regulation of ROS production, scavenging, and transcriptional control that protects cells against oxidative injury and preserves physiological homeostasis.

#### 4.6.3 Evidence from recent systematic reviews and meta-analyses supporting oxidative stress pathway

Recent meta-analytic evidence underscores that oxidative stress biomarkers are strongly linked with worse COVID-19 outcomes. In a 2023 free radical biology/meta-analysis (via Ovid), lower levels of glutathione, altered SOD, and elevated AOPPs were significantly different between survivors and non-survivors, with glutathione levels below ∼327.2 μmol/mL associated with ∼3.12-fold higher mortality risk (adjusted hazard ratio) after adjustment for confounders (Neves et al., 2023). Another recent systematic review and meta-analysis found that ischemic-modified albumin, a marker of oxidative injury, is elevated in COVID-19 patients, while total thiol levels are reduced, especially in critically ill cases, reinforcing redox imbalance as a disease-modifying axis (Mousavi et al., 2025).

Genetic and longitudinal evidence further strengthen the mechanistic link between redox biology and COVID outcomes. A 2025 MDPI systematic review of glutathione S-transferase (GST) polymorphisms notes that variants in these antioxidant enzyme genes may predispose to sustained oxidative stress and influence long COVID trajectories, bridging inherited antioxidant capacity to clinical progression (Villegas Sánchez et al., 2025). A 2024 longitudinal/supplementary evidence study reported that systemic oxidative stress predicts post-COVID syndrome, aligning with gene–protein associations (e.g., MBL2, ADAMTS13) that connect oxidant stress to endothelial dysfunction and chronic sequelae (Valente Coronel et al., 2025). Finally, a focused 2024 review on MPO emphasized its role as a central effector of oxidative tissue injury across diseases (including lung injury/COVID), lending mechanistic support to the GWAS-inferred causal role of MPO pathways in severe COVID-19 (Georgieva et al., 2023).

### 4.7 Comparative strength of biomarkers: evidence from this review

While oxidative stress biomarkers provide important insights, the strength of evidence varies considerably (Table 7). For example, catalase activity shows inconsistent patterns, with some studies reporting increased activity in severe cases, whereas others find no significant change, limiting its prognostic reliability. Similarly, SOD levels are variably reported as elevated, decreased, or unchanged across patient cohorts, suggesting that SOD activity may be context-dependent (e.g., variant-specific or stage-specific). In contrast, HO-1 activity and NOS-derived nitrate levels demonstrate more consistent associations with severity and mortality, indicating stronger prognostic value.

**TABLE 7:** Comparative strength of biomarkers: Evidence from this review.

Biomarker category	Strong evidence	Inconsistent evidence	Weak/Preliminary evidence
Enzymatic Activity	HO-1 (severity, ICU, mortality) NOS activity (nitrate/nitrite: mortality predictor)	Catalase activity (increased in some ICU cases, no change in others) SOD levels (elevated, decreased, unchanged across studies)	MPO, XO activity (limited data, not validated prognostic)
Genetic Polymorphisms	SOD2 rs4880 (Val16Ala: reproducible severity link) HMOX1 promoter variants (rs13057211: higher mortality)	GSTM1/GSTT1 null genotypes (mortality risk in some studies, protective in others) GSTP1 Ile105Val (conflicting evidence)	PRDX, TXN/TXNR, NQO1 variants (few or no reproducible studies)
Micronutrient Cofactors	Selenium deficiency (mortality, immune impairment) Zinc deficiency (worse prognosis, immune dysfunction)	Copper, manganese (changes in severe cases, inconsistent)	Iron (complex role; deficiency and overload harmful, indirect evidence)
Oxidative Damage Biomarkers	MDA, 8-isoprostanes, nitrotyrosine, protein carbonyls (consistently linked to ARDS, ICU, mortality)	—	—
miRNAs (Oxidative Stress–Responsive)	miR-21, miR-146a (reproducible links to oxidative stress, severity, immune regulation)	miR-155 (variable evidence across cohorts)	Other miRNAs (preliminary findings, not yet validated)

HO-1, heme oxygenase-1; NOS, nitric oxide synthase; SOD, superoxide dismutase; MPO, myeloperoxidase; XO, xanthine oxidase; SOD2, superoxide dismutase 2; HMOX1, heme oxygenase 1; GST, glutathione S-transferase; GSTM1/GSTT1, glutathione S-transferase Mu 1/Theta 1; GSTP1, glutathione S-transferase Pi 1; PRDX, peroxiredoxin; TXN/TXNR, thioredoxin/thioredoxin reductase; NQO1, NAD(P)H quinone dehydrogenase 1; MDA, malondialdehyde; ARDS, acute respiratory distress syndrome; miRNA/miR, microRNA.

With respect to genetic polymorphisms, evidence for GST variants remains inconsistent: while some studies link GSTM1/GSTT1 null genotypes to higher mortality, others report no significant associations or even potential protective effects at population level. By comparison, SOD2 rs4880 and HMOX1 promoter variants show more reproducible links to severity and outcomes, although replication in larger, multi-ethnic cohorts is still needed.

Beyond enzymatic activity and genetics, micronutrient cofactors also display variable evidence. Selenium and zinc deficiencies consistently correlate with worse outcomes, impaired antioxidant defences, and increased mortality, positioning them as stronger prognostic biomarkers with translational potential. In contrast, evidence for copper and manganese is less consistent, with some studies suggesting altered levels in severe COVID-19, but without reproducible prognostic value. Iron status remains complex: both deficiency and overload appear detrimental, but its role is often confounded by inflammation and anaemia of chronic disease, limiting direct prognostic interpretation.

Finally, oxidative stress damage biomarkers such as MDA, 8-isoprostanes, nitrotyrosine, and protein carbonyls demonstrate the most reproducible associations with severity, respiratory failure, ICU admission, and mortality. Emerging evidence also highlights the role of oxidative stress–responsive microRNAs (e.g., miR-21, miR-155, and miR-146a), which regulate redox-sensitive pathways and inflammatory cascades during COVID-19. Among these, miR-21 and miR-146a currently have the strongest supporting evidence, showing reproducible associations with oxidative stress status, disease severity, and immune dysregulation across multiple cohorts, suggesting their potential as complementary prognostic biomarkers alongside enzymatic and genetic indicators. These biomarkers consistently reflect cumulative oxidative damage and correlate with poor outcomes across multiple cohorts, strengthening their utility as prognostic indicators.

### 4.8 Clinical translation of oxidative stress biomarkers in COVID-19

To enhance the translational perspective of this review, we classified the biomarkers discussed into three categories: (i) validated biomarkers consistently associated with severity or mortality across multiple cohorts, (ii) assay-ready biomarkers that can be measured using standardized clinical or research assays, and (iii) exploratory candidates that remain insufficiently validated. This stratification clarifies which biomarkers are closest to clinical implementation and which require further replication in larger studies.

#### 4.8.1 Validated in large or multiple patient cohorts

Several studies have shown that serum HO-1 levels correlate with severity and outcomes in ARDS, interstitial lung disease exacerbations, and COVID-19 (Hara et al., 2022). Reviews have documented elevated oxidative stress biomarkers in multiple cohorts of COVID-19 patients vs. controls, supporting reproducibility across populations (Tsermpini et al., 2022).

#### 4.8.2 Assay-ready/clinically measurable biomarkers

Commercial ELISA kits exist, and HO-1 measurement has already been applied in clinical studies of lung injury (Nagasawa et al., 2020) and COVID-19 (Hara et al., 2022). Some biomarkers, such as lipid peroxidation products (e.g., MDA, 8-isoprostanes), antioxidant enzyme activities (e.g., SOD, catalase) are already part of oxidative stress panels in research labs and may be adapted for clinical use.

#### 4.8.3 Exploratory biomarkers

Although studied in COVID-19 and other conditions, results are inconsistent and insufficient for clinical adoption. For instance, Jerotić et al. showed that SOD2 rs4880 and GPX1 rs1050450 polymorphisms did not confer clear COVID-19 risk in their cohort (Jerotic et al., 2022). Other antioxidant gene polymorphisms or less-studied redox regulators remain in the exploratory stage, lacking replication or cohort-level validation. Table 8 summarizes the translational readiness of oxidative stress–related biomarkers, integrating enzymatic, genetic, and micronutrient determinants of COVID-19 prognosis.

**TABLE 8:** Clinical utility and translational potential of oxidative stress biomarkers in COVID-19.

Biomarker	Clinical translational status
HO-1 (Heme Oxygenase-1)	Validated in multiple cohorts; assay-ready (ELISA kits available)
MDA (Malondialdehyde)	Validated in several cohorts; assay-ready (TBARS/LC-MS)
8-isoprostanes (8-iso-PGF2α)	Validated; ELISA and LC-MS/MS assays available
Nitrotyrosine	Validated but less standardized; ELISA/LC-MS/MS feasible
Protein Carbonyls, 8-OHdG, 4-HNE	Exploratory; assays exist but limited replication
SOD2 rs4880	Validated genetic biomarker; genotyping is assay-ready
HMOX1 rs13057211	Emerging validated biomarker; needs replication, but genotyping feasible
NOS3 polymorphisms (e.g., rs2070744)	Exploratory/Population-specific; requires larger validation
Selenium & Zinc	Validated and assay-ready; easily measurable, clinically actionable
PON1 activity	Assay-ready, but more diagnostic than prognostic
TXN/TXNRD, PRDXs, GST variants	Exploratory; not validated in large cohorts

Abbreviations: HO-1: Heme Oxygenase-1; MDA: malondialdehyde; 8-iso-PGF2α: 8-isoprostaglandin F2α; 8-OHdG: 8-hydroxy-2′-deoxyguanosine; 4-HNE: 4-hydroxy-2-nonenal; SOD2: Superoxide Dismutase 2; HMOX1: Heme Oxygenase 1 gene; NOS3: Nitric Oxide Synthase 3; PON1: Paraoxonase 1; TXN: thioredoxin; TXNRD: thioredoxin reductase; PRDXs: Peroxiredoxins; GST: Glutathione S-transferase.

## 5 Clinical disappointments and translational barriers of using oxidative/antioxidatives as prognostic biomarkers

The measurement of OS biomarkers has faced numerous challenges and disappointments, which have limited their clinical utility and hindered progress in antioxidant-based therapies. A major issue lies in the choice of biomarkers and biological systems, as no single biomarker has been universally validated to reliably reflect OS across different diseases. Many commonly used biomarkers, such as F_2_-isoprostanes, exist in multiple forms (free *versus* esterified), and measuring only one form in a single biological fluid may lead to misleading interpretations. Furthermore, disease-related changes in diet, lifestyle, or medication can alter biomarker levels independently of OS, introducing significant confounding factors. Pre-analytical and analytical pitfalls also contribute to inconsistency, since improper sample collection, storage, and processing can lead to artificial increases in biomarker levels, while many assays lack sufficient sensitivity and specificity. In addition, variability in laboratory protocols and occasional scientific misconduct have undermined reproducibility and reliability of results. These technical and methodological weaknesses have been mirrored in clinical research, where large randomized trials of antioxidant supplementation (e.g., vitamin E, vitamin C, β-carotene, or N-acetylcysteine) have yielded contradictory or mostly negative outcomes, with some studies even suggesting potential harm, such as increased risk of heart failure or mortality. These disappointing results may stem from late or inappropriate timing of supplementation, use of inadequate dosages, or reliance on antioxidants that may not target the relevant reactive species and can even act as pro-oxidants under certain conditions. Importantly, while OS biomarkers are elevated in many pathological conditions, their presence often reflects a secondary response rather than a primary cause, making it difficult to establish causal links between OS and disease progression (Giustarini et al., 2009).

One of the key disappointments stemmed from the non-specific nature of many OS biomarkers. Elevations in MDA, 8-OHdG, or nitrotyrosine are not exclusive to COVID-19 but are commonly observed in other inflammatory and metabolic conditions such as diabetes, obesity, and cardiovascular disease (Giustarini et al., 2009). Micronutrient cofactors also produced inconsistent prognostic and therapeutic outcomes. For instance, while low Se levels were associated with severe COVID-19 in some cohorts, Se supplementation did not consistently translate to clinical benefit. Similar discrepancies were observed with Zn, a popular supplement during the pandemic, which often failed to improve key outcomes despite theoretical benefits in immunity and oxidative regulation (Patel et al., 2021).

## 6 Strategies to overcome the limitations and challenges in measuring oxidative stress biomarkers

To overcome the limitations and challenges in measuring OS biomarkers, several strategies have been proposed to improve both research reliability and clinical translation. First, there is a critical need for the standardization of biomarker selection and analytical methodologies, as the lack of consensus on which biomarkers best reflect oxidative damage has resulted in conflicting data. Establishing validated biomarker panels, rather than relying on a single parameter, may provide a more comprehensive assessment of oxidative status across different diseases. Second, rigorous pre-analytical protocols must be implemented, including careful control of sample collection, storage, and preparation, to prevent artificial formation or degradation of biomarkers that can distort results. The use of quality control samples processed in parallel with study specimens is essential to ensure reproducibility. Third, advanced and specific analytical techniques, such as high-performance liquid chromatography coupled with mass spectrometry (HPLC-MS/MS), should replace older, less reliable assays like TBARS, which lack specificity. Fourth, clinical trial design should be improved by selecting patients with documented evidence of elevated OS, rather than including heterogeneous populations where effects may be diluted. Moreover, antioxidant interventions must consider dose, timing, and bioavailability, as well as the compartmentalization of reactive species, since mismatches between the antioxidant used and the reactive species targeted are a major reason for trial failures. Finally, the development of integrative approaches, combining OS biomarkers with genetic, metabolic, and imaging data, may offer better mechanistic insights and disease-specific predictive value. These strategies, supported by strict validation and quality assurance, could substantially enhance the reliability of OS measurement and pave the way for more effective therapeutic applications (Giustarini et al., 2009).

## 7 Conclusion

Oxidative stress plays a central role in the pathophysiology and prognosis of COVID-19, linking viral replication, immune dysregulation, and multi-organ damage. Enzymatic antioxidants, genetic variants, micronutrient cofactors, oxidative damage biomarkers, and miRNAs each provide unique windows into disease dynamics. However, the strength of evidence supporting these biomarkers varies considerably.

Biomarkers with strong and consistent evidence include HO-1 activity, NOS-derived nitrate/nitrite levels, selenium deficiency, SOD2 rs4880 polymorphism, oxidative damage biomarkers such as MDA, 8-isoprostanes, nitrotyrosine, and miRNAs such as miR-21 and miR-146a, which reproducibly associate with oxidative stress regulation, severity, and immune dysregulation. These biomarkers repeatedly associate with ICU admission and mortality, making them prime candidates for translational studies and clinical prognostic models.

In contrast, several biomarkers demonstrate inconsistent evidence, such as catalase activity, SOD activity, GST polymorphisms, and miR-155, where findings diverge depending on patient population, study design, or disease stage. These inconsistencies underscore the need for larger, standardized, and multi-ethnic studies. Other biomarkers, including MPO, XO activity, PRDXs, TXN/TXNR, NQO1 variants, and other miRNAs, remain weak or preliminary, with insufficient data to confirm prognostic utility.

Moving forward, integrating well-validated biomarkers, including oxidative stress–responsive miRNAs, into clinical practice could enable earlier identification of high-risk patients, better allocation of healthcare resources, and tailored therapeutic strategies. At the same time, acknowledging variability and knowledge gaps ensures realistic expectations for translation. Future research should focus on harmonizing biomarker assays, validating findings across diverse cohorts, and testing whether nutritional or pharmacological interventions targeting these redox pathways can improve outcomes.
